# New insights into checkpoint inhibitor immunotherapy and its combined therapies in hepatocellular carcinoma: from mechanisms to clinical trials

**DOI:** 10.7150/ijbs.70691

**Published:** 2022-03-28

**Authors:** Haoer Jin, Sha Qin, Jiang He, Juxiong Xiao, Qingling Li, Yitao Mao, Luqing Zhao

**Affiliations:** 1 Department of Pathology, Xiangya Hospital, Central South University, Changsha, Hunan, China; and Department of Pathology, School of Basic Medical Science, Xiangya School of Medicine, Central South University, Changsha, Hunan, China.; 2 Center for Molecular Medicine, Xiangya Hospital, Central South University, Changsha, Hunan, China.; 3 Department of Radiology, Xiangya Hospital, Central South University, Changsha, Hunan, China.; 4 National Clinical Research Center for Geriatric Disorders, Xiangya Hospital, Central South University, Changsha, Hunan, China.

**Keywords:** Hepatocellular carcinoma (HCC), Tumor microenvironment (TME), Programmed cell death protein 1 (PD-1), Cytotoxic T lymphocyte 4 (CTLA-4), Immune checkpoint inhibitors (ICIs)

## Abstract

Hepatocellular carcinoma (HCC) is one of the most lethal tumors in China and worldwide, although first-line therapies for HCC, such as atezolizumab and bevacizumab, have been effective with good results, the researches on new therapies have attracted much attention. With the deepening research on tumor immunology, the role and operation mechanism of immune cells in the tumor microenvironment (TME) of HCC have been explained, such as programmed cell death protein 1 (PD-1) binding to ligand could cause T cell exhaustion and reduce IFN-γ T cell secretion, cytotoxic T lymphocyte 4 (CTLA-4) and CD28 mediate immunosuppression by competing for B7 protein and disrupting CD28 signal transduction pathway, which also lays the foundation for the development and application of more new immune checkpoint inhibitors (ICIs). The biological behavior of various immune checkpoints has been proved in HCC, such as PD-1, programmed cell death ligand 1 (PD-L1), CTLA-4 and so on, leading to a series of clinical trials. Currently, FDA approved nivolumab, pembrolizumab and nivolumab plus ipilimumab for the treatment of HCC. However, the treatment of ICI has the disadvantages of low response rate and many side effects, so the combination of ICIs and various other therapies (such as VEGF or VEGFR inhibition, neoadjuvant and adjuvant therapy, locoregional therapies) has been derived. Further studies on immune checkpoint mechanisms may reveal new therapeutic targets and new combination therapies in the future.

## Background

According to the latest statistics in 2021, HCC was the sixth most commonly diagnosed cancer and the third leading cause of cancer death globally 1. Compared with other regions, the incidence rate of HCC is highest in East Asia. Among those countries, Mongolia has the highest incidence (93.7 per 100,000) 2. With the progress of science and medicine, the treatment of HCC has a great development. Treatments of HCC are mainly based on tumor stage. Researchers have put forward many kinds of cancer staging systems, of which Barcelona Clinic HCC (BCLC) staging system is most widely recognized and clinically used 3. According to the early-stage HCC patients, they are primarily treated by surgery, such as hepatic resection (HR), liver transplantation, ablation, etc. For the intermediate-stage HCC patients, transarterial chemoembolization (TACE) is the treatment of choice. Before 2016, there was only sorafenib in the systematic therapy of patients with advanced hepatocellular carcinoma (aHCC). With the deepening of research on the treatment of HCC in recent years, tumor immunotherapy provides a new direction for the treatment 4-8.

Immunotherapy may be a hotspot in the treatment of HCC in the next few years. In this paper, we studied the role of immune cells in the TME of HCC, summarized the immune checkpoints and their ligands in the field of HCC, concluded the clinical progress of ICIs, and discussed and compared the current use of the combined ICIs strategy. Finally, we review the future development of ICIs in HCC.

## The immune network in TME of HCC

**Figure 1:**
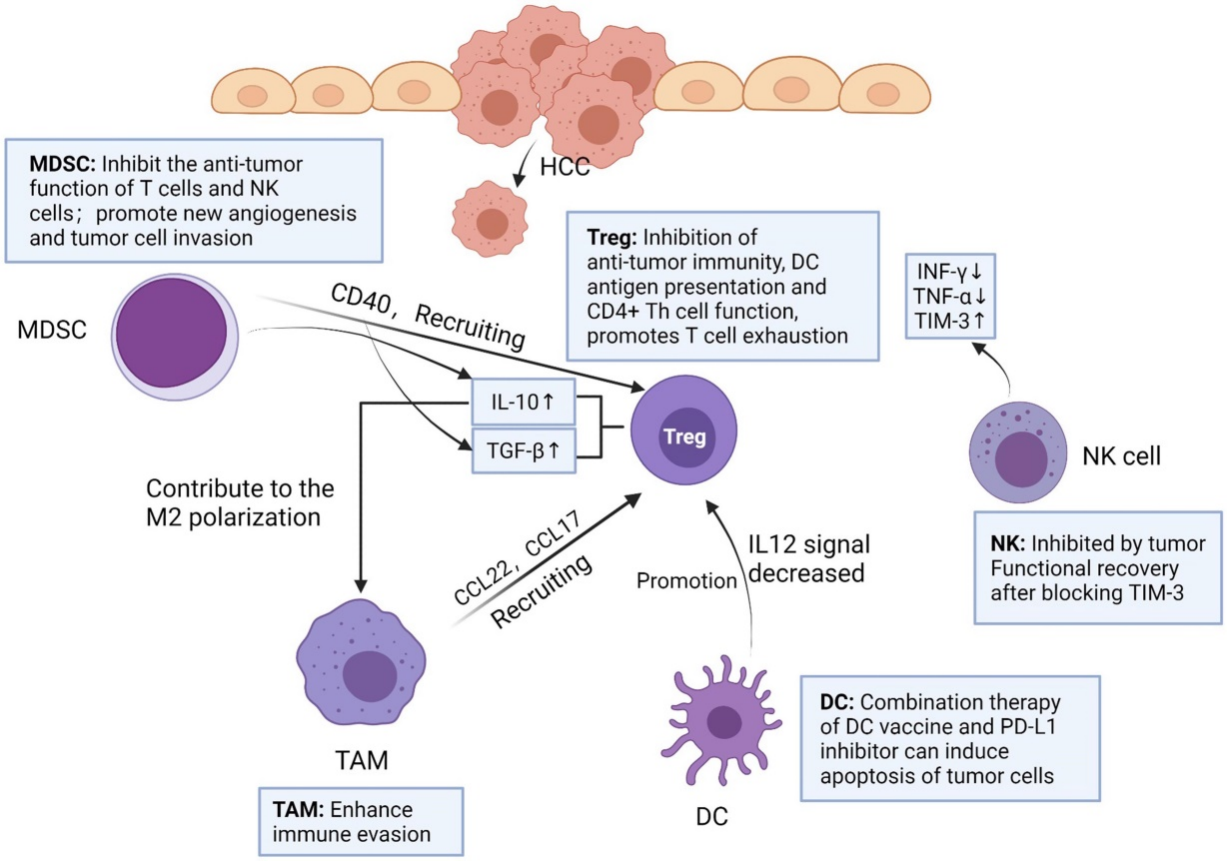
** Interaction and functions of immune cells in HCC tumor microenvironment.** MDSC can secrete TGF-β, IL-10 and recruit Treg by expressing CD40. TAM can produce chemokines CCL22 and CCL17, and recruit Treg to transfer to cancer sites. IL-10 secreted by Treg promotes M2 polarization of TAM. DC promotes Treg by reducing IL-12 secretion. The number of NK cells in tumor tissues of HCC patients decreased, and the levels of IFN-γ and TNF-α secreted by NK cells decreased, but NK cells increased the expression of TIM-3. After blocking TIM-3, the function of depleted NK cells recovered rapidly. Created with BioRender.com.

Immune cells play an important role in TME. Among them, the innate immune system of liver is very necessary in the process of resisting tumor invasion, mainly including natural killer (NK) cells, Kupffer cells (KCs) and dendritic cells (DCs) 9. In addition, tumor-infiltrating lymphocyte (TIL) is an important component of solid tumors, which is the host's attempt to mediate anti-tumor response 10. However, more and more experimental data showed that TIL in HCC could not produce effective anti-tumor immunity to inhibit tumor progression 11. Therefore, immunosuppressive cells play a major role in tumor immune tolerance and immune evasion in HCC, including tumor-associated macrophages (TAMs), regulatory T cells (Tregs) and myeloid-derived suppressor cells (MDSCs) in HCC tumor microenvironment 12-14 **(Figure 1)**.

### Natural killer cell (NK)

NK cells are one of the major lymphocytes in liver immunity, accounting for 30-50% of the total number of lymphocytes in the liver 15. NK cells can express a variety of cytokines to regulate immune function. In HCC, NK cells are exhausted, but they highly express immune checkpoints and secrete immunosuppressive cytokines, such as TGFβ, IL-10 and IFN-γ 16, 17. This involves a general mechanism in liver diseases, referring to MDSC-mediated NK cell impairment. After the NK cells of HCC patients were cultured with M-MDSCs *in vitro*, the cytotoxicity of NK cells was significantly reduced, and the secretion of IFN-γ was also reduced 18. Notably, experimental data showed that the frequency of NK cells in peripheral blood and liver were positively correlated with the survival time of patients with HCC 19. NK cells and CD8^+^ T cells in tumors have increased T cell immunoglobulin and mucin domain-containing protein 3 (TIM-3) expression 20. Zhang PF et al found that circUHRF1 was highly expressed in HCC tissue and exosome derived from HCC. Besides, they demonstrated that circUHRF1 could up-regulate the expression of TIM-3 through sponge miR-449c-5p, resulting in NK cell exhaustion. Finally, they concluded that the exocrine circUHRF1 secreted by HCC cells reduces the effectiveness of anti-PD-1 therapy by inducing NK cell exhaustion 21. Tan, S et al. used flow cytometry to analyze the single cells infiltrated by HCC, and found that NK cells were significantly reduced, while TIM-3 expression was increased. After blocking TIM-3, the depleted NK cells function quickly recovered, thereby inhibiting tumor growth 22. Taken together, simultaneous use of TIM-3 and/or PD-1 inhibitors can reverse T cell depletion and reduce tumor growth.

### Dendritic cell (DC)

DCs can present antigen, control T cell differentiation and regulate T cell response 23. DCs initiate T cells against tumor associated antigens (TAA) involved in HCC progression 9. DCs can promote tumor growth in HCC patients. There are mainly two mechanisms, namely, inducing tolerance to tumor antigens and inhibiting T cell function by releasing cytokines or expression immune checkpoint ligands. HCC cells can induce immature differentiation of DCs by secreting immunosuppressive factors such as IL-10 and VEGF. Immature DCs promote tumor tolerance by inducing CD8^+^ Treg and inhibit the function of other effector T cells 24. In HCC tumor immune microenvironment, DCs promote Tregs and impair T cells proliferation by reducing IL-12 secretion 25. Based on the characteristics of DCs, researchers had conducted several clinical trials to assess the efficacy of DC vaccines in patients with HCC. For example, in patients with advanced HCC, mature autologous DCs with isolated pulse of liver tumor cell line lysate (HepG2) were injected intravenously, and it was found that HCC patients had good tolerance to the vaccine 26. However, the overall results of DC vaccination alone have not significantly improved the therapeutic effect. In a recent study, researchers used DC vaccine combined with PD-L1 inhibitor in a mouse model of HCC 27. Compared with the single treatment, the combined treatment of DC vaccine and PD-L1 inhibitor can induce higher levels of tumor cell apoptosis by increasing the infiltration of cytotoxic T cells against tumor cells.

### Kupffer cell (KC) and Tumor-associated macrophage (TAM)

Liver macrophages are composed of KCs and recruited macrophages. KCs are resident groups of liver macrophages, which mainly exist in the hepatic sinusoidal. KCs account for about 20% of non-solid cells in the liver 28. During host defense, KCs perceive pathogens and coordinate inflammatory responses, including phagocytosis, antigen processing and presentation, and secretion of proinflammatory mediators 25. KCs are considered to be HCC-specific TAMs 29.

**Figure 2:**
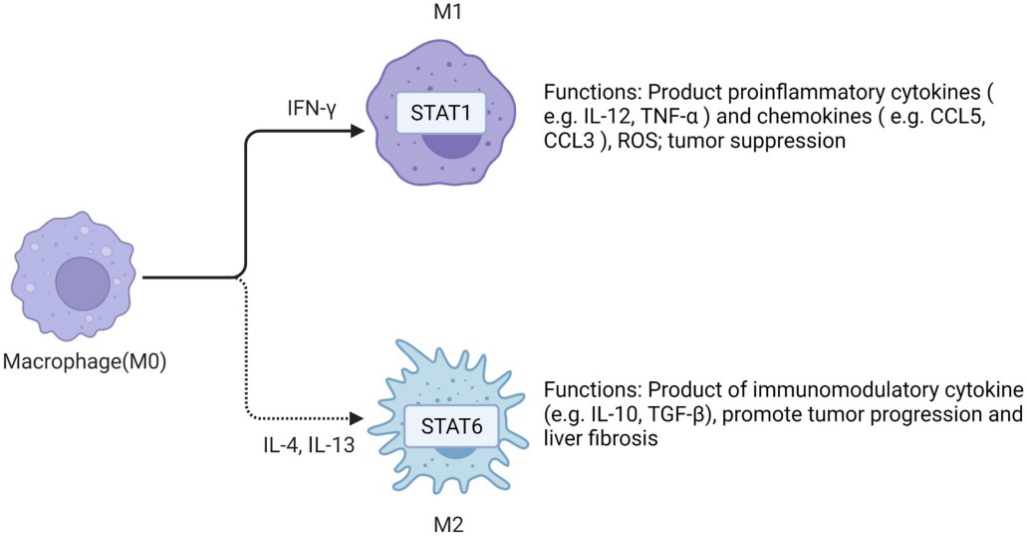
** Macrophages have two polarization directions.** IFN-γ promotes M1 macrophage polarization by activating STAT1. IL-4 and IL-13 promote M2 macrophage polarization by activating STAT6. M1 and M2 macrophages have different functions in the liver. Created with BioRender.com.

**Figure 3:**
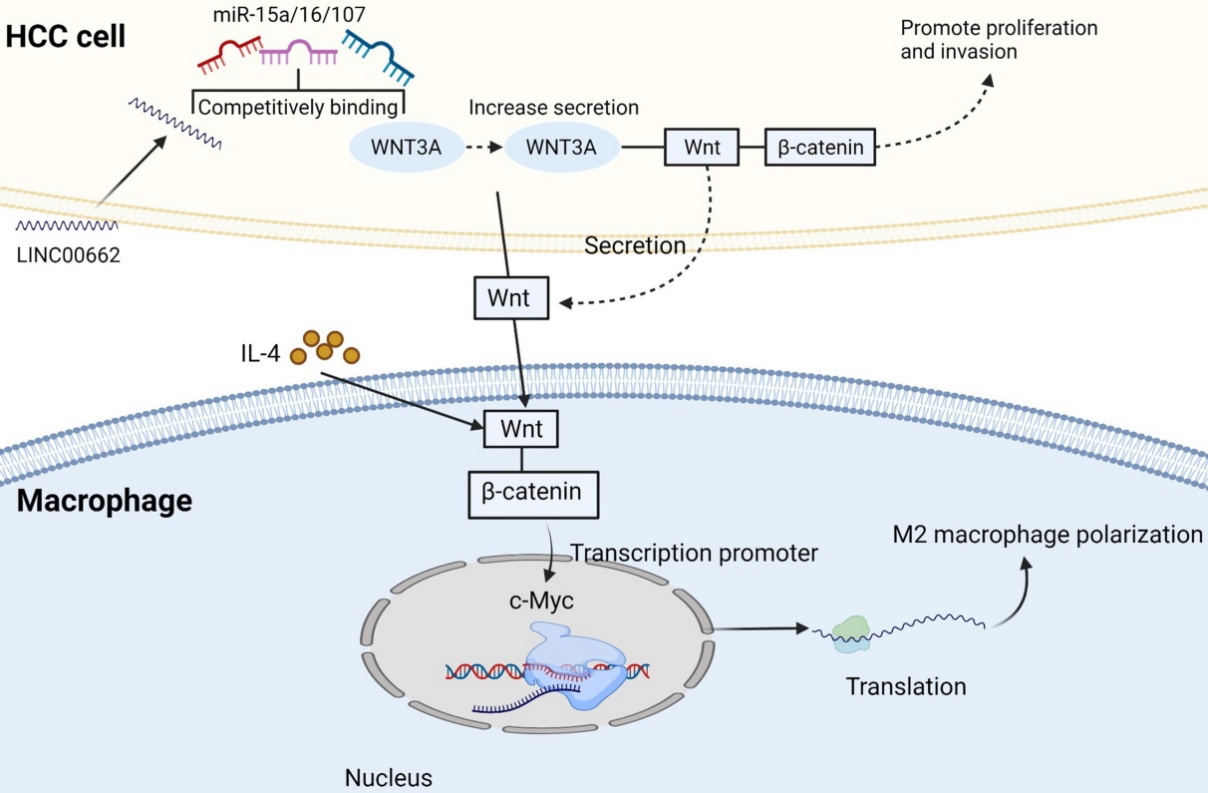
** M2 macrophage polarization related pathways.** IL-4 activates Wnt/β-catenin signaling pathway in macrophages, resulting in up-regulation of c-Myc expression. C-Myc can promote M2 polarization. Long non-coding RNA LINC00662 up-regulated the expression of Wnt-3a by binding to miR-15a/16/107, activated Wnt/β-catenin signal, and promoted the proliferation and growth of liver cancer cells *in vivo*. Meanwhile, WNT3a induces M2 macrophage polarization by paracrine. Created with BioRender.com.

TAMs in HCC microenvironment are closely associated with poor prognosis. Studies have demonstrated that TAMs repolarization to anti-tumor phenotype promotes tumor regression. In general, macrophages can polarize into M1 or M2 macrophages, and stimulation of factors such as INF-γ can activate M1 polarized macrophages. M1 polarization can produce immune stimulating factors and promote inflammation, such as IL-12 or TNF-α 30. However, it was found that M2-polarized macrophages were more similar to TAMs, and the former could be activated by Th2 cytokines, namely IL-13 and IL-4 31 **(Figure 2)**. Yang Y et al. first reported that Wnt ligands derived from tumor cells promote polarization of M2 macrophages through canonical Wnt/β-catenin signaling, leading to tumor growth, migration, metastasis and immunosuppression in HCC 32. More and more researchers have focused on blocking Wnt secretion and/or activating of Wnt/β-catenin signaling in tumor cells. For example, long noncoding RNA LINC00662 was demonstrated to be able to up-regulate the expression of WNT3A by binding to miR-15a/16/107, activate Wnt/β-catenin signaling, and further promote the proliferation and growth of HCC cells *in vivo*. At the same time, WNT3A induces the polarization of M2 macrophages in a paracrine manner 32-34 **(Figure 3)**.

TAMs can produce a variety of chemokines and interact with other immune cells. For example, IL-1 β released by M2-TAMs can promote EMT and metastasis of HCC cells under hypoxia through HIF-1α/IL-1β/TLR4 axis 35. CCL22 and CCL17 can recruit Tregs to move to the cancer site, which enhances immune evasion 36. It has been reported that the inhibitory cytokine IL-10 secreted by Tregs contributes to the M2 polarization of TAMs. In addition, Tregs could promote M2 polarization of TAMs by inhibiting CD8^+^ T cell-IFN-γ axis 37. This suggests that there may be a positive feedback loop between TAMs and Tregs, providing a new perspective for the immunosuppressive effect of cancer. It was reported that the number of Tregs in HCC patients increased 38, and the proportion and absolute number of Foxp3^+^ CD25^+^ CD4^+^ Treg subsets increased significantly.

### Regulatory T cell (Treg)

Yang Y et al. examined 235 surgical specimens from HCC patients and found that CD8^+^ T cells were exhausted and Tregs were accumulated in tumors. Multiple markers (LAG-3, TIM-3, CTLA-4, PD-1 and other immune checkpoints) co-expressed in T cells of HCC, thus blocking these immune checkpoints may reverse exhausted T cells 39. Tregs are a kind of CD4^+^ T cells that can be divided into two groups: natural regulatory T cells (nTregs) and induced-to-adjust T cells (iTregs). Both kinds of Tregs express Foxp3 40. nTregs are present in the thymus, induced by autoantigens and inhibited by intercellular contact. Unlike nTregs, iTregs are originated from peripheral naïve CD4^+^ T cells induced by cytokines specific to the tumor microenvironment, such as IL-10, TGF-β 41. TGF-β and IL-10 promote the differentiation of iTregs by up-regulating the expression of Foxp3 and CTLA-4, leading to the proliferation and migration of tumor cells, resulting in poor prognosis of patients 42. With the progress of tumor, tumor cells secrete TGF-β in an autocrine manner, forming positive feedback. Although there are differences between nTregs and iTregs, it is difficult to distinguish these two types *in vivo*, and nTregs and iTregs are rarely distinguished in the literature describing Treg in TME 43.

Tregs can maintain immunosuppressive state in tumor microenvironment by releasing inhibitory cytokines such as transforming growth factor (TGF)-β, IL-10 and IL-35 38, 44. Tregs can inhibit anti-tumor immunity, DC antigen presentation and CD4^+^ T helper (Th) cell function. Shi C et al found that Tregs promoted the migration and invasion of Hepa1-6 cells *in vitro* and *in vivo* through EMT. In the wound healing experiment, Hepa1-6 cells treated with Tregs-CM had faster healing effect, which was also detected by transwell migration assay. In addition, after using TGF-β1 neutralizing antibody in Treg-CM, the EMT ability and colony formation ability of Hepa1-6 cells were significantly decreased. Therefore, it can be speculated that the consumption of TGF-β1 can block the effect of Tregs-induced EMT, which may be a potential reason for promoting the invasive migration of HCC cells 45. It is worth noting that IL-10 and IL-35 secreted by Tregs can directly induce the expression of inhibitory receptors on CD8^+^ cytotoxic T lymphocytes (CTLs) in tumors and promote T cells exhaustion by regulating the BLIMP1 inhibitory receptor axis in CD8^+^ T CTLs 46.

### Myeloid-derived suppressor cells (MDSC)

MDSC, as a heterogeneous kind of immature myeloid cells, are divided into two major MDSC subsets: mononuclear MDSC (M-MDSC) and polymorphonuclear MDSC (PMN-MDSC) 47. Both subgroups have been verified to have immunosuppressive effects. PMN-MDSCs are the main components of MDSCs in tumor-bearing mice, and have the same morphology and characteristics as neutrophils. They inhibit antigen-specific CD8^+^ T cells by producing high levels of reactive oxygen species (ROS) 47, 48. However, in the surrounding tissues of HCC patients, the number of M-MDSCs is more than that of PMN-MDSCs 49. M-MDSCs have similar morphology and characteristics to monocytes and are considered to have stronger immunosuppressive activity in tumor microenvironment than PMN-MDSCs in some cases 50. M-MDSCs produce a large number of NO, arginase-1 and immunosuppressive cytokines. Since these molecules have longer half-life period, M-MDSCs can effectively inhibit nonspecific T cell response without the requirement for close contact between M-MDSCs and T cells 51. Liu M et al. reported that activated hepatic stellate cells (HSC) can activate P38 MAPK signaling in M-MDSCs and then promote the accumulation and function of M-MDSCs through chromatin regulation mediated by CCAAT-Enhancer-Binding Protein Beta (C/EBPβ) 49. However, Liu M did not point out which cytokines secreted by HSCs caused this phenomenon. Another article expounded that HSCs could secrete IL-6 to promote the proliferation of MDSCs (mainly M-MDSCs), and make MDSCs secrete more immunoregulatory enzymes, such as inducible nitric oxide synthase (iNOS) and arginine 1 52.

In conclusion, MDSCs have two main functions: on the one hand, it promotes tumor progression by inhibiting the anti-tumor function of T cells and NK cells; on the other hand, MDSCs also promote new angiogenesis and tumor cell invasion 53. Previous studies have shown that MDSCs from HCC patients can inhibit the cytotoxicity and cytokine secretion of NK cells when cultured with NK cells *in vitro* 54. MDSCs can express high levels of PD-L1, and can also recruit Tregs by expressing CD40. In addition, MDSCs can also express a variety of immunosuppressive cytokines, such as TGF-β, IL-10 55.

During the development of HCC, immune cells have different effects in different pathways. Focusing on immune cells, researchers have developed a variety of immunomodulators and used them in cancer therapy, leading to a breakthrough in the study of ICIs 56. ICIs act on T cells, mainly cytotoxic T lymphocytes, and activates downstream immune pathways to achieve anti-tumor purposes. Antibodies aiming at molecular CTLA-4, PD-1, PD-L1 and so on have been used in many kinds of tumors, and therapeutic effects have been achieved 57-60. Below, we discuss their roles in HCC and focus on their strategies of immunotherapy.

## Systemic treatment in HCC

### Immune checkpoints in HCC

#### PD-1 and PD-L1/PD-L2

**Figure 4:**
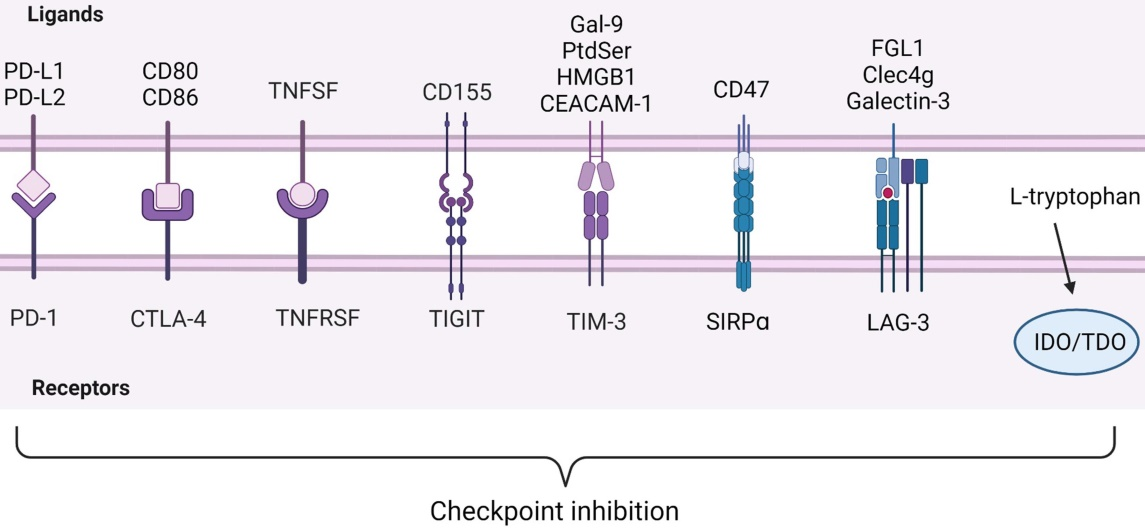
** ICIs observed in immunocyte. Below are inhibitory receptors expressed on the surface of immune cells.** The ligands of these receptors expressed by tumor cells lie above the image. IDO and TDO are enzymes in immune cells that react with L-tryptophan. Inhibitory receptor-ligand interactions leading to immune escape of cancer cells are called checkpoint inhibition. Created with BioRender.com.

Under the normal physiological condition, the human immune system protects the host from disease invasion through the cooperation of various immune cells. However, growing evidences indicate that tumors utilize a variety of methods such as immune checkpoints in the immune system to evade anti-tumor immune response 61, 62 **(Figure 4)**. Only a small number of patients have improved symptoms. Understanding the drug resistance mechanism is necessary to improve the efficacy of anti-PD-1^63^. PD-1, also known as CD279, is an immunosuppressive molecule 64. In HCC, Binding of PD-1 to ligand triggers downregulation of T cell receptor, inhibits T cell activation and cytokine release. It regulates the immune system and promotes self-tolerance by down-regulating the response of the immune system to human cells and inhibiting the inflammatory activity of T cells. PD-1 is expressed on activated T cells, B cells and macrophages and negatively regulates immune response more widely than CTLA-4 57, 64-67.

The ligands for PD-1 are PD-L1 (also known as B7-H1 or CD274) and PD-L2 (also known as B7-DC or CD273). They belong to the B7 family of type I transmembrane protein receptors. PD-L1 is widely expressed in hematopoietic as well as non-hematopoietic cells 68. Binding of PD-L1 to PD-1 on T cells leads to dephosphorylation of T cell receptor, resulting in reducing T cell proliferation and activity. Tumor cells make use of this mechanism to escape immune surveillance 69. Different from PD-L1, PD-L2 was initially thought to be predominantly expressed in APCs, such as DCs, macrophages and bone marrow-derived mast cells. However, recent studies have shown that PD-L2 can be expressed by various immune cells and tumor cells under microenvironment stimulation. Some studies demonstrate that PD-L2 expression in tumor cells is a predictive factor for the clinical efficacy of anti-PD-1 mAb 70, 71.

T cell exhaustion could be defined as a state of differentiation observed during chronic infection with persistent antigen and chronic T cell receptor (TCR) stimulation 72, 73. With the rapid development of oncology, in-depth study of this concept is of great significance for checkpoint blockade. Compared with normal T cells, exhausted T cells consistently express multiple inhibitory receptors, such as PD-1, TIM-3 74. When T cells were exhausted seriously, the co-expression of TIM-3 and PD-1 could be observed, suggesting that the clinical prognosis of HCC was poor 75, 76. Studies have shown that blocking PD-1 inhibitory receptors *in vivo* can restore depleted T cells by acting on the TME 77.

A recent study found that OTU domain-containing ubiquitin aldehyde-binding protein 1 (OTUB1) regulates the activation of CD8^+^ T cells and NK cells through IL-15 78. Meanwhile, OTUB1 regulates PD-L1 abundance through the endoplasmic reticulum-associated degradation (ERAD) pathway. Degradation of PD-L1 increased and content decreased due to lack of OTUB1. OTUB1 deficiency leads to HCC cells to become more sensitive to T cell mediated cytotoxicity, inhibits tumor growth and enhances anti-tumor immunity in mice by regulating PD-L1 79, 80.

**Figure 5:**
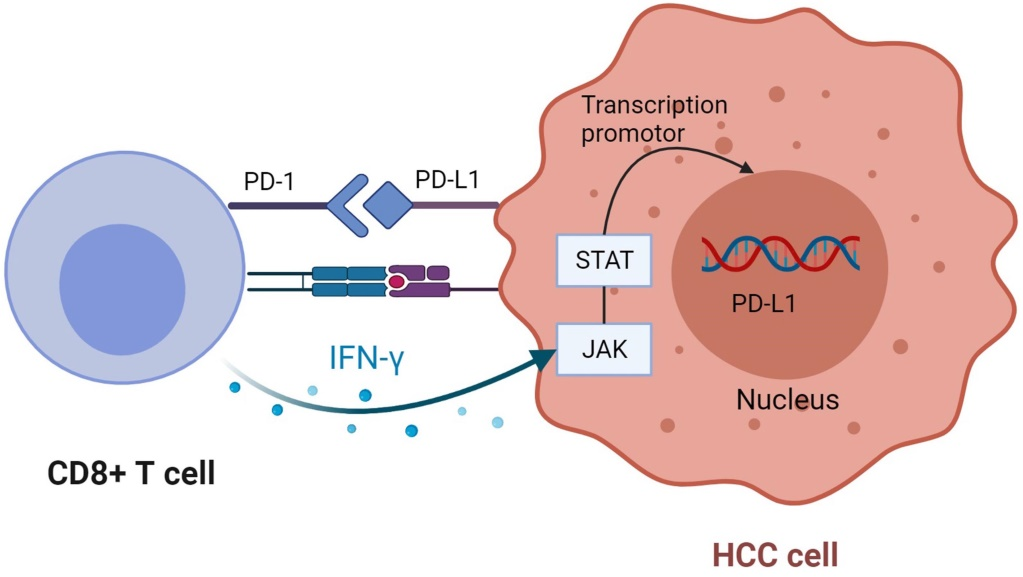
** CD8 + T cells upregulate PD-L1 expression in tumor cells.** CD8 + T cells secrete IFN-γ after recognizing tumor antigens, bind to the corresponding receptors on tumor cells, and up-regulate the expression of PD-L1 through JAK-STAT pathway. Created with BioRender.com.

When PD-1 binds to ligand, it inhibits T cell response and reduces T cell secretion of IFN-γ 61. Studies have found that IFN-γ secreted by CD8^+^ T cells after tumor antigen recognition can upregulate PD-L1 expression. The subtype 2 protein kinase D induced by IFN-γ is a vital factor regulating PD-L1. Inhibition of PKD2 activity can lead to down-regulation of PD-L1 expression and promote a strong anti-tumor immune response 61, 63, 68, 81. Studies have indicated that IFN-γ induces upregulation of PD-L1 expression in cancer cells mainly through JAK-STAT pathway. By analyzing the data in TCGA, it is concluded that PD-L1 expression is positively correlated with IFN-γ characteristics 82, 83 **(Figure 5)**. In addition to cancer cells, PD-L1 also exists on the surface of its derived exosomes, and overexpression of IFN-γ or PD-L1 increased the level of PD-L1 in exosomes 84, 85. Classical double-positive T (DPT) cells, known as naive T cells in the thymus, show strong immune response after stimulation, which improves the prognosis of HCC patients. The unique distribution of PD-1^+^ DPT in HCC verifies this conclusion 86.

Although PD-1 ligand PD-L1 has been studied deeply, the detailed mechanism of another PD-1 ligand PD-L2 is still unclear. Compared with PD-L1, PD-L2 has weaker immunosuppressive effect in animal models commonly used in tumor immunology research 70. But the effect of low PD-L2 expression as a measure of immune infiltration is better than PD-L1. PD-L2 is superior to PD-L1 in evaluating anti-tumor immunity and IFN-γ signal transduction 87, 88. A study shows that PD-L2 and other B7-28 family molecules can be used as prognostic indicators of immunotherapy. The activation of JAK-STAT pathway can up-regulate the expression of PD-L2, and blocking this activation can increase the production of IFN-γ 89. Some studies have reported that PD-L2 positivity and overexpression are associated with adverse prognosis in terms of OS for HCC 90.

#### CTLA-4 and CD80/CD86

**Figure 6:**
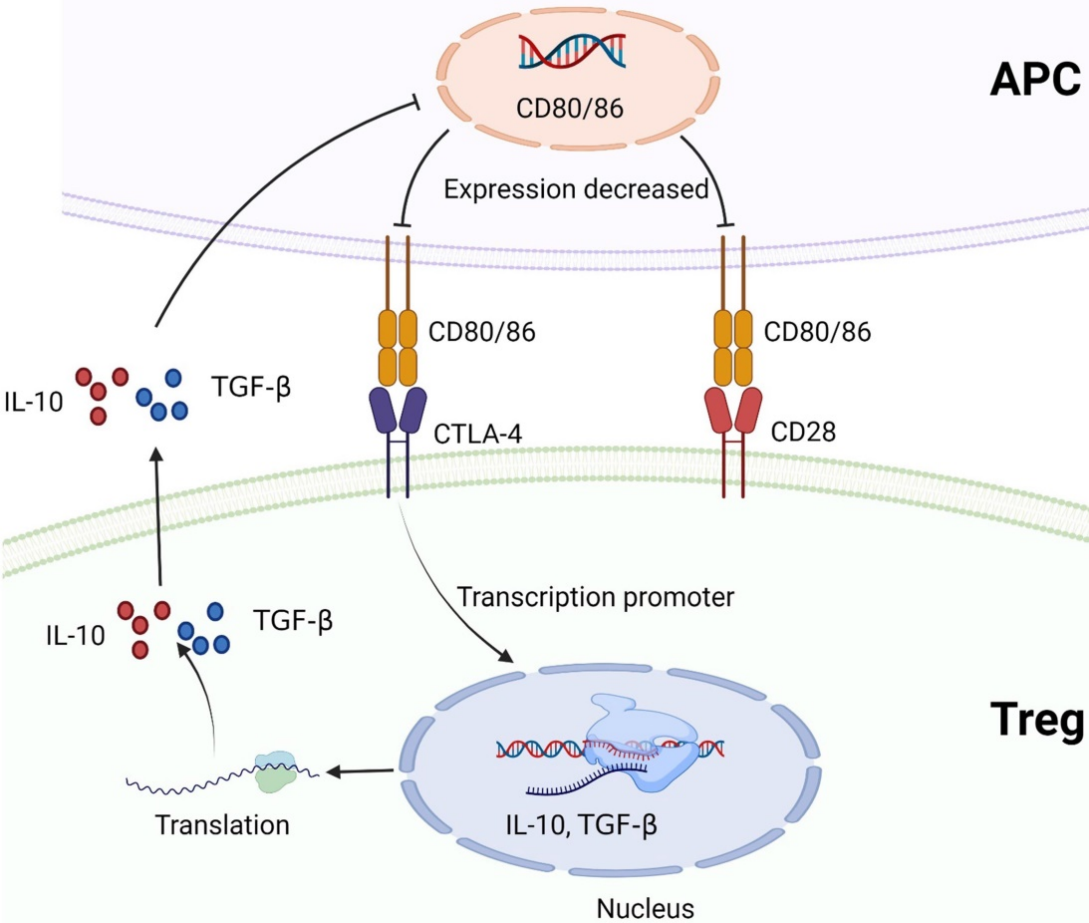
** CTLA-4 and CD28 compete to bind to B7 protein (CD80/CD86).** CTLA-4 promotes Treg to produce cytokines that inhibit B7 expression, such as IL-10 or TGF-β, and reduces the expression of B7 molecules on APC. Created with BioRender.com.

CTLA-4 plays a negative regulatory role in the immune system, mainly expressed on Treg cells. The expression of CTLA-4 is up-regulated when T cells are activated, and the degree of T cell inflammatory response is reduced 91, 92. TGF-β1 upregulates PD-1 and CTLA-4 expression through TGF-βR/CaN/NFATc1 signaling in a dose-dependent manner, thereby enhancing tumor immune escape in HCC 93. CD28 and CTLA-4 are homologous glycoproteins of the immunoglobulin superfamily. Contrary to CTLA-4, the activation of CD28 signaling pathway by B7 leads to the production of T cell cytokines and the increase of T cell proliferation 94. CTLA-4 mediates immunosuppression by competing with CD28 for B7 protein and disrupting CD28 signaling pathway. After binding to B7 protein, CTLA-4 inhibits the destruction of proximal TCR signaling and central supramolecular cluster (c-SMAC) in immune synapses. B7/BB1 is a cell surface protein called CD80, and B7-2 (CD86) is another CTLA-4 ligand **(Figure 4)**. These ligands were highly expressed in antigen presenting cells (APC) 95, 96. In addition, CTLA-4 reduces the expression of B7 molecules on APC by producing cytokines such as IL10 or TGF-β that inhibit B7 expression 97, 98 **(Figure 6)**. CTLA-4 also acquires ligands by transendocytosis (TE) and APC to degrade them in recipient cells 98, 99. However, Yang Y et al. reported that B-1a cells in adult mouse spleen can also express CTLA-4. They established CKO mouse model and elucidated that CTLA-4 inhibits B-1a cell activation 100.

CTLA-4 targeted therapy for HCC faces two challenges: poor efficacy and adverse reactions. Therefore, safer and more effective CTLA-4 targeted therapy is needed in medicine to improve the benefit-risk situation. Kvarnhammar AM et al. discovered that ATOR-1015, a CTLA-4 x OX40 bispecific antibody, could activate T cells by blocking CTLA-4 and significantly enhance antitumor response. Interestingly, they also found that ATOR-1015 enhanced the anti-tumor response to anti-PD-1 treatment. So they thought ATOR-1015 could be used in combination with anti-PD-L1 in the future 101.

#### Other Immune checkpoints

In the related studies of HCC, only PD-1 and CTLA-4 were well understood. In recent years, many new immune checkpoints have emerged and have achieved research results in other cancer fields. Although there are few studies on HCC, it is likely to be used in combination with existing treatment regimens for HCC in the future.

##### TNFSF/TNFRSF

Most immune checkpoints inhibit immune cell activation, but tumor necrosis factor receptor superfamily (TNFRSF) as T cell costimulatory receptor promotes immune cell activation^102^** (Figure 4)**. Previous studies have shown that TNFRSF members can be highly expressed by activated CD4^+^ and CD8^+^ effector T cells and nonactivated Tregs, thereby promoting and inhibiting adaptive immunity, respectively^103^, ^104^. In effector T cells, the activation of TNFRs can promote the proliferation and differentiation of CD4^+^ and CD8^+^ lymphocytes, and also participate in the apoptosis of T cells 105. Although the function of TNFRSF is poorly understood in Treg cells, these receptors release the necessary signaling molecules to maintain thymus development and proliferation 106. It was found that after T cell activation *in vitro*, only the expression of GITR was up-regulated in TNFRSF members of Tregs, and the expression levels of TNFR2, 4-1BB or OX40 on resting Tregs were low. The agonists of TNFR2, 4-1BB, GITR, and DR3 need to activate classical NF-κB pathways to co-stimulatory Tregs. TNFRSF co-stimulation in Tregs is also conducive to the expression of IL-4, IL-5, IL-13 and IL23p19 immune molecules 107. In recent years, more and more studies focus on OX40 and CD27, which are also members of TNFRSF. Clinical trials of agonists targeting OX40 and CD27 with tumors are under way, combination of OX40 and CD137 induces T cell proliferation in a short time, which may bring new directions for the treatment of HCC 108, 109. The ligand of CD27 (TNFRSF7) is CD70, which affects innate immune system by increasing IFN-γ production by NK cells. Studies have found that CD27/CD70 signal transduction is very important for anti-cancer immunity, and the combination of agonistic CD27 antibodies and PD-1 blocking has the highest curative effect in solid tumor clinical trials 108. Although there are few studies on CD27 and HCC, it has shown good anti-tumor effect on most solid tumors.

##### CD155/TIGIT

T cell immunoreceptor with immunoglobulin and ITIM domain (TIGIT) is a potential new target for cancer immunotherapy in addition to CTLA-4 and PD-1. It can effectively inhibit innate immunity and adaptive immunity 110, 111** (Figure 4)**. TIGIT is expressed on TILs of various human tumors, and its expression on TILs is closely related to PD-1 expression. TIGIT expression was detected in all tumor-derived PD1^Hi^ CD8^+^ T cells 76. In addition, compared with TIM-3 and LAG-3, TIGIT is also expressed on CD8^+^ PD-1 int TIL of patients with high PD-1 expression. Compared with single PD-1 blocking, the combined blocking of TIGIT and PD-1 obviously enhances the proliferation of CD8^+^ TILs 112, 113. TIGIT mainly has two ligands: CD155 (PVR) and CD112 (PVRL2, nectin-2), which have the highest affinity with CD155 110. The expression of CD155 was significantly up-regulated in tumor cells of various cancers and correlated with poor prognosis 114. TIGIT can produce immunosuppressive effect on CD8^+^ T cells of HCC through CD155/TIGIT signal transduction, such as PI3K, MAPK and NF-κB signaling pathways, reduce the contents of IFN-γ, tumor necrosis factor-α, and IL-17A, and increase the content of IL-10. It was found that the ratios of p-AKT/AKT and p-ERK/ERK in CD8^+^ T cells co-cultured with wild type HCC cells was significantly lower than that of CD155 knockdown cells, which was reversed by blocking TIGIT 115. It can be speculated that CD155 overexpression in HCC cells may escape host immune response by upregulating TIGIT on TIL. According to this feature, Ge Z et al. studied the effect of TIGIT combined with PD-1 blocker on CD8^+^ T cells in hepatocellular carcinoma. They found that combination of TIGIT and PD-1 significantly increased IFN-γ production by blocking enhanced CD8^+^ T cell function *in vitro* 112. When regulating the functions of T cells and NK cells, it mediates signal transduction through interaction with costimulatory immune receptor CD226 (DNAM-1), inhibitory checkpoint receptor TIGIT and CD96, which is a very important immune ligand 116. TIGIT can be detected on activated CD4^+^ T cells, CD8^+^ T cells and Foxp3^+^ Treg cells. TIGIT was highly expressed in CD4^+^ T cells and Treg cells in HCC patients. In addition, the expression of TIGIT was positively correlated with the expression of alpha fetoprotein (AFP) 117.

##### TIM-3 and Gal-9/PtdSer/HMGB1/CEACAM-1

TIM-3 is a kind of checkpoint receptor, recent studies have found that inhibition of TIM-3 enhances the anti-tumor effect of PD-1 blockers. TIM-3 was first discovered in 2002 and was initially thought to be a receptor expressed on IFN-γ-producing CD4^+^ and CD8^+^ T cells 75, 118. Based on the TIM-3 expression, CD8^+^ T cells could be classified into three distinct subpopulations: PD1^Hi^, PD1^Int^ and PD1^-^, but TIM-3 expression was only limited to PD1^Hi^ CD8^+^T cells 119. Through investigating the ability of CD8^+^ TILs to produce cytokines based on PD-1 expression and measuring cytokines, researchers observed that the frequency of IL-2-producing PD1^Hi^ CD8^+^ T cells was under-regulation, as well as IFN-γ and TNF-α, while the frequency of IL-10-producing was increased in PD1^Hi^ CD8^+^ T cells 76. The characteristics of less cell metabolic activity and reduced production of pro-inflammatory cytokines suggest that PD1^Hi^ CD8^+^ T cells are in T cell exhaustion 120.

TIM-3 has four ligands, including galactose lectin 9 (Gal-9), phosphatidylserine (PtdSer), high mobility group box-1 protein (HMGB1) and carcinoembryonic antigen-related cell adhesion molecule 1 (CEACAM-1) 75** (Figure 4)**. The combination of TIM-3 and Galectin-9 can mediate the apoptosis of effector T cells through the calcium-calpain-caspase-1 pathway and increase the production of IFN-γ in NKs 121-123. PtdSer is a molecule exposed to apoptotic cells and binds to Tim-3 on DCs to mediate uptake and cross-presentation of apoptotic cells 124. HMGB1 is mainly released by stressed or dying cells, responsible for transporting nucleic acids to internal vesicles, which is necessary to promote innate immune responses to pathogens and tumors. The binding of HMGB1 to TIM-3 interferes with this process, thereby weakening the antitumor effects of DNA vaccines and cytotoxic chemotherapy and inhibiting innate and antitumor immune responses 125. The co-expression of TIM-3 and CEACAM1 was limited to a small fraction of activated T cells, and the function of TIM-3 is independent of CEACAM1. TIM-3 and CEACAM1 can promote inhibitory signaling pathways in T cells 126. Tan S et al found that TIM-3 is the most abundant immune checkpoint receptor expressed on tumor NK cells in HCC. As an endogenous ligand, PtdSer induces Tim-3 phosphorylation and inhibits NK cell function by interfering with PI3K/Akt/mTOR pathway 22. Cytokines in TME can induce the expression of TIM-3 in HCC cells, including IL-4, TGF-β and IL-6. Hepatocyte-Tim-3 receptor activates NF-κB phosphorylation, thereby stimulating IL-6 secretion and STAT3 phosphorylation 127. This process promotes tumor growth and increases metastasis of HCC cells by enhancing epithelial-mesenchymal transition (EMT) 128. It can be concluded that TIM-3 is not only expressed in immune cells, but also in HCC cells. These results show that TIM-3 may have other functions in addition to suppressing immune response. All studies further illustrated the important role of TIM-3 as a new participant in HCC progression and a promising target for HCC immunotherapy.

##### CD47/SIRPɑ

Under physiological conditions, CD47/SIRPɑ axis plays a protective role in preventing macrophages from clearing hematopoietic cells **(Figure 4)**. However, under pathological conditions, cancer cells may use the axis to escape immune surveillance 129. IL-6 secreted by TAMs upregulates CD47 expression in hepatocellular carcinoma cells, which inhibition of STAT3 signal destroys this effect. The results showed that there was a positive correlation between STAT3 phosphorylation and CD47 expression in tumor cells. Anti-phagocytosis mediated by CD47 may reduce the efficacy of TACE in hepatocellular carcinoma cells treated with chemotherapy drugs 130. Du K et al. drew on the specific expression characteristics of Glypican-3 (GPC3) in HCC and the inhibitory effect of CD47 on macrophages to generate a novel bispecific antibody (BsAb): GPC3/CD47 BsAb. They demonstrated that GPC3/CD47 BsAb has potent antitumor activity against tumor cells expressing double antigens 131. Du J et al. also designed a new anti-tumor therapy. They used the principle of CD47 surface functionalization (Exos^CD47^) to make exosomes effectively avoid the phagocytosis of mononuclear phagocyte system (MPS) to construct exosomes, and encapsulated ferroptosis inducer (Erastin, Er) and photosensitizer (Rose Bengal, RB) into exosomes to induce iron death of HCC cells 132. These provide antibody design information for the future development of innovative immunotherapy.

##### IDO/TDO

Tryptophan is an essential amino acid, which is metabolized mainly through the kynurenine pathway. Indoleamine 2,3-dioxygenase (IDO) and tryptophan 2,3-dioxygenase (TDO or TDO2) are the starting enzymes and key enzymes to catalyze this pathway **(Figure 4)**. It has been found that the removal of tryptophan in the microenvironment can inhibit the proliferation and activity of T cells. Kynurenine is an immunosuppressive molecule that inhibits the proliferation and activity of T cells and natural killer cells 133. TDO promotes EMT through the Kyn-AhR pathway, contributing to the invasion and metastasis of HCC and leading to poor prognosis 134. Chinnadurai R et al. found in the study that canine uridine inhibited T cell proliferation in the absence of PD-L1 Ig, and the inhibitory effect of the combination of the two on T cell proliferation was better than that of them alone. In addition, PD-L1 Ig and canine uridine synergistically inhibited IFN-γ secretion 135. High expression of IDO in tumor cells can promote the expansion, recruitment and activation of MDSCs 136. Clinical trials of IDO1 inhibitor BMS-986205 combined with Nivolumab as first-line or second-line therapy for HCC patients are underway (ClinicalTrials.gov Identifier: NCT03695250). Therefore, canine uridine pathway and small molecule inhibitors of IDO are expected to become potential tumor immunotherapy drugs. However, the TME of HCC is extremely complex. In order to determine the immune regulation pathway and interaction between IDO and other ICI, it is necessary to further study the mechanism and function.

##### LAG-3/FGL1

Lymphocyte activating gene 3 (LAG-3) is an immunosuppressive receptor, and major histocompatibility complex II (MHC-II) is a typical ligand 137. However, recent studies have shown that fibrinogen-like protein 1 (FGL1) is another major LAG-3 functional ligand **(Figure 4)**. FGL1 is a protein secreted by hepatocytes, as a mitogen to promote hepatocyte proliferation. However, FGL1 is usually low in normal hepatocytes, while it is significantly increased in HCC cells. The final results support FGL1/LAG-3 pathway as a potential target for immune escape and cancer immunotherapy 138. Galectin-3 and C-type lectin-like domain containing-4g (Clec4g) have been proved to interact with LAG-3 and inhibit T cell function, but the mechanism is not completely related to LAG-3 137, 139, 140. The levels of LAG-3, FGL1, PD-L1 and CD8^+^ T cells in 143 HCC patients were evaluated by multiple immunofluorescence. It was found that the density of LAG-3^+^ cells was positively correlated with the level of FGL1 and the expression of PD-L1, but the correlation between LAG-3^+^ cells and the former was significantly stronger than that of the latter. In addition, the number of CD8^+^ T cells was positively correlated with PD-L1 level, but negatively correlated with FGL1 expression 141. Therefore, the expression of LAG-3 may represent a biomarker for unfavorable prognosis of HCC. Wang J et al. studied the therapeutic mechanism of Oxysophocapine on HCC. In this experiment, compared with anti-IL-6R treatment alone, Oxysophocarpine combined with anti-IL-6R did not show more effective inhibition on the expression of FGL1, P-JAK2 and P-STAT3, indicating that Oxysophocarpine inhibits IL-6-mediated JAK2/STAT3 signaling activation in HCC cells to downregulate FGL1 expression, thereby improving the anti-LAG-3 therapeutic effect 142.

### ICI clinical trials in HCC

After sorafenib first-line treatment, the FDA has approved three other therapies, including nivolumab, pembrolizumab and nivolumab plus ipilimumab in HCC treatment 4.

#### Nivolumab: anti-PD-1 monoclonal antibody

A phase I/II study investigated the role of immunotherapeutic agent nivolumab in disease progression in patients with advanced HCC receiving at least one systemic treatment, including sorafenib, or intolerance to sorafenib 143. Based on the findings, the FDA accelerated approval of nivolumab as a second-line treatment for advanced HCC in September 2017 144 **(Table 1)**.

A randomized study showed a comparison between nivolumab (NIVO) and sorafenib (SOR) as first-line treatment in patients for aHCC 145. A total of 743 patients with aHCC participated in this study. The shortest follow-up period was 22.8 months. The median survival time of patients after nivolumab treatment was longer than that after sorafenib treatment (15.2 vs 13.4 months, HR 0.85 [95% CI: 0.72-1.02], P=0.075). Although the predefined threshold of statistical significance was also not met (HR 0.84, P=0.0419), NIVO showed better therapeutic effect than SOR in patients with aHCC.

Choi WM et al. evaluated the clinical results of nivolumab in patients with Child-Pugh B HCC through a real-world cohort study 146. The study involved a total of 203 patients, 132 with Child-Pugh A and 71 with Child-Pugh B. The longest follow-up period was 37 months, with a median of 5.6 months. 146 patients died during the follow-up period and 150 patients deteriorated after nivolumab treatment. ORR in Child-Pugh B group was remarkably lower than that in Child-Pugh A group (2.8% vs 15.9%), but disease control rate (DCR) was lower than that in Child-Pugh A group (22.5% vs 42.4%). After statistics, researchers found that the OS of Child-Pugh A group was longer than Child-Pugh B group (42.9 vs. 11.3 weeks; 95% confidence interval [CI], 2.15-4.24; p < 0.001), as well as median progression-free survival (PFS) (7.4 vs. 6.0 weeks; 95% CI, 1.22-2.29; p = 0.014). In this study, most HCC patients are caused by HBV infection, so the universality of this study may be limited and more cases are needed to improve the results.

In the phase 2/3 trial CheckMate 040 Cohort 5 (NCT01658878), patients with aHCC and Child-Pugh B cirrhosis received intravenous nivolumab 240 mg every 2 weeks in order to explore whether it is suitable for Child-Pugh B aHCC patients. The results showed that ORR was 12% (95% CI 5-25%), disease control rate was 55% (95% CI 40-69%), and median duration of response (DOR) was 9.9 months (95% CI 9.7-9.9). Thus, the investigators concluded that, nivolumab showed good therapeutic effect and safety for Child-Pugh B aHCC patients 147.

#### Pembrolizumab: anti-PD-1 monoclonal antibody

In 2018, pembrolizumab was approved by the FDA for patients with HCC previously treated with sorafenib 148. It was evaluated a randomized, double-blind, phase III trial (KEYNOTE-240). A total of 413 subjects in this study were characterized by ineffective treatment of sorafenib. The patients were randomly divided into pembrolizumab group and control group according to the ratio of 2:1. The experimental group received intravenous injection of 200 mg pembrolizumab every 3 weeks while the control group received the same amount of saline placebo for at least 35 cycles. Median OS was 13.9 months (95% CI, 11.6-16.0 months) in the pembrolizumab group and 10.6 months (95% CI, 8.3-13.5 months) in the control group (HR, 0.781; 95% CI, 0.611-0.998; P = 0.0238). In addition, the median PFS of pembrolizumab group was slightly longer than that of placebo group (3.0 vs 2.8; 95% CI; 0.570-0.904; P = 0.0022). Although the median PFS of the two groups was close, the Kaplan-Meier curve showed that the efficacy of pembrolizumab was better in some patients after long-term use. The study did not meet its prespecified statistical dual end points of improving PFS and OS with pembrolizumab, but the results show that pembrolizumab has good therapeutic effect in patients.

A study aimed to evaluate the tolerance, safety and efficacy of lenvatinib plus pembrolizumab in unresectable hepatocellular carcinoma (uHCC) has recently obtained experimental results (ClinicalTrials.gov identifier NCT03006926) 149. In this Ib multicenter open label study of 100 patients, lenvatinib plus pembrolizumab yielded a definite response rate (46% by mRECIST; 36% by RECIST v1.1) per independent imaging review (IIR), median PFS of 9.3 months (by mRECIST; 8.6 months by RECIST v1.1) per IIR, and median OS of 22.0 months. This suggested that lenvatinib plus PD-1 inhibition and pembrolizumab monoclonal antibody lead to good antitumor effect. Preclinical data indicated that the immunoregulation of lenvatinib enhanced the activity of pembrolizumab monoclonal antibody, leading to increased tumor sensitivity.

#### Ipilimumab: anti-CTLA-4 monoclonal antibody

In phase 1/2 studies, PD-1 inhibitors have shown therapeutic efficacy as second-line therapies for HCC 143, 150. The combination of nivolumab and ipilimumab has higher safety, expected objective response rate and sustained response. The checkmate 040 randomized clinical trial (ClinicalTrials.gov identifier NCT01658878) 151. In this experiment, patients with advanced HCC who had previously received sorafenib treatment were randomly divided into three groups and treated with different schemes, and then the safety of different schemes was evaluated. Based on those results, one group of schemes (4 doses nivolumab 1 mg/kg plus ipilimumab 3 mg/kg every 3 weeks then nivolumab 240 mg every 2 weeks) get the best experimental results. The objective response rate of the group was 32% (95% CI, 20% to 47%). In the experiment, the group did not meet the median (range) response duration (8.3 to 33.7+). Of the 49 patients involved in the study, 46 had any-grade treatment-related adverse events and one died of pneumonia of grade 5. Taken together, this treatment received accelerated approval in USA. Similar results were obtained in another study (ClinicalTrials.gov identifier NCT03222076) 152. Researchers found that nivolumab plus ipilimumab could also be used during perioperative period for resectable HCC.

Although immune checkpoints have brought new development directions for HCC treatment, this treatment has many adverse reactions and poor efficacy. To explore the effective treatment for patients with advanced HCC who failed to block PD-1 pathway, Wong JSL et al. designed a combination therapy of CTLA-4 and PD-1 blockers, namely that all patients needed to receive ipilimumab 1 mg/kg with nivolumab 3 mg/kg or pembrolizumab 2 mg/kg scheduled every 3 weeks 153. About half of patients had primary drug resistance. The median follow-up was 37.7 months (95% CI: 32.8 to 42.7). According to statistical analysis, ORR reached 16% and approximately 40% of patients achieved clinical benefits in this experiment. The median DOR was 11.5 months (95% CI, 2.76 to 30.3). The median OS reached 10.9 months (95% CI, 3.99 to 17.8). These data provide a new research direction for solving the urgent clinical problems of anti-PD-1/L1 refractory advanced HCC patients.

### Combination therapy related to ICIs in HCC

#### Adverse effect of ICIs

Use of ICIs is the most important breakthrough in cancer therapy in the past decade. ICIs, including anti-CTLA-4, anti-PD-1 and anti-PD-L1 antibodies, are increasingly being used in various malignancies, but a series of new immune-related adverse events (irAEs) follow. When irAEs are serious, it can even endanger life 154. A study in the United States showed that the recurrence rate of the same irAEs was 28.8% when cancer patients were treated with the same ICI again 155. This undoubtedly brings a series of new challenges in clinical treatment. It was found that compared with the single use of anti-CTLA-4 monotherapy, the use of anti-PD-1 or anti-PD-L1 monotherapy and combination therapy would lead to more severe irAEs. In the experiment of nivolumab combined with ipilimumab in patients with advanced HCC (ClinicalTrials.gov Identifier: NCT01658878), the results showed that the incidence of adverse reactions of nivolumab and ipilimumab regimen was higher than previously reported nivolumab monotherapy 151. In addition, the recurrence rate of colitis was higher than those of other irAEs 155 **(Table 2)**. Clinically, the adverse reactions of ICI include autoimmune endocrine diseases, colitis, pituitary inflammation, hepatitis, pneumonia, etc. 156. In an experiment on the clinical index of long-term survival of advanced hepatocellular carcinoma treated with immune checkpoint, the patients were divided into short-term survival group (31 patients) and long-term survival group (5 patients). Interestingly, two patients (40 %) in the long-term survival group had grade 3 or 4 immune-related adverse events (IrAEs-3/4), but no IrAEs-3/4 was found in the short-term survival group. So researchers believe that IrAEs-3/4 may be associated with long-term survival in patients with advanced HCC 157. However, there are only five long-term survival groups in this study, and the sample is not representative. In-depth study of irAEs can deepen our understanding of autoimmune diseases and provide new ideas and clues for the treatment of autoimmune diseases.

### Combination of VEGF or VEGFR inhibition and ICIs

#### Background and preclinical studies

HCC is a kind of highly vascularization tumor with abundant arterial blood flow. Patients with advanced HCC often have excessive blood vessels and obvious vascular abnormalities 158, 159. Therefore, antiangiogenic drugs have good therapeutic prospects. All FDA approved systemic therapies for HCC are molecular targeted therapies that target VEGF signaling pathways leading to anti-angiogenesis 160. HCC identified a new subtype, designated as macrotrabecular-massive HCC (MTM-HCC). It had frequent satellite nodules and macrovascular and/or microvascular infiltration, with high invasiveness. Angiogenesis activation is a significant feature of MTM-HCC, in which angiopoietin 2 and VEGF-A are overexpressed 161. VEGF-A and pro-inflammatory cytokines induce endothelial cells to express FasL, thereby obtaining the ability to kill CD8^+^ T cells, but Treg cannot be killed. The pharmacological function of VEGF-A increases the number of CD8^+^ cells in tumor and inhibits tumor growth 158, 162. Among the approved MKIs for the treatment of HCC, sorafenib's immunoregulatory effect has been the most widely studied. A large number of studies have elucidated that sorafenib enhances anti-tumor immunity by increasing the M1 polarization of TAMs, enhancing the infiltration and function of CD4^+^ and CD8^+^ T cells, inhibiting the number of Tregs and reversing the function of MDSCs in tumor microenvironment 163. In addition, regorafenib induced polarization of M1 macrophages to promote the proliferation and activation of CD8^+^ T cells 164, 165.

Although immune checkpoint blockers have been approved by FDA for HCC treatment, the use of PD-1 blockers is only beneficial for a small fraction of patients with hepatocellular carcinoma. Similarly, combined VEGF or VEGFR inhibition and ICI in patients with HCC can also have a good therapeutic effect 166, 167. The mouse model of HCC was established and the results showed that the growth of primary tumor was significantly delayed by specific blocking of vascular endothelial receptor 2 by mouse antibody, but the survival time did not change obviously. However, after dual anti-PD-1/VEGFR-2 treatment, the growth of primary tumors was inhibited, and the survival rate was estimated to be twice as high as the original. After further investigation of the mechanism, researchers found that the upregulation of PD-L1 in HCC depends on VEGFR-2 blocking in endothelial cells, which is mediated by IFN-γ expression in endothelial cells 166. A new study shows that anti-PD-1 treatment with levatinib enhances anti-tumor immune response in HCC by activating immune pathways, reducing Tregs infiltration and inhibiting TGF-β signaling 168.

#### Clinical studies

The network meta-analysis (NMA) of 14 low-bias risk trials showed that the combination of atezolizumab and bevacizumab had the best clinical efficacy in the first-line treatment of advanced HCC patients 169. A therapeutic strategy of unresectable HCC had previously been studied using the combination of atezolizumab with bevacizumab (a kind of VEGF inhibition), which also showed encouraging antitumor activity and safety 170. The IMbrave150 (ClinicalTrials.gov identifier NCT03434379) trial compared 1200 mg of atezolizumab plus 15 mg/kg of bevacizumab intravenously every 3 weeks, 400 mg of sorafenib orally twice daily. Statistical analysis showed that the overall survival of patients in atezolizumab-bevacizumab group was significantly longer than that in sorafenib group. The 6-month and 12-month survival rates of the atezolizumab-bevacizumab group were 84.8% (95% CI, 80.9-88.7) and 67.2% (95% CI, 61.3-73.1), respectively. The 6-month and 12-month survival rates of sorafenib group were 72.2% (95% CI, 65.1-79.4) and 54.6% (95% CI, 45.2-64.0), respectively. In addition, progressive survival in atezolizumab-bevacizumab was also markedly longer than in sorafenib (median, 6.8 months [95% CI, 5.7-8.3] vs. 4.3 months [95% CI, 4.0-5.6]).

Several other trials are assessing the clinical efficacy of VEGFR blockers combined with ICI. The efficacy and safety of camrelizumab (an anti-PD-1 monoclonal antibody) plus apatinib (a vascular endothelial growth factor [VEGFR]-2 tyrosine kinase inhibitor) were evaluated in patients with aHCC^171^. Oral apatinib 250 mg was given daily. At the same time, patients received intravenous injection of camrelizumab 200 mg (weight ≥ 50 kg) or 3 mg/kg (weight < 50 kg) every 2 weeks. Of a total of 190 patients, 70 received first-line setting and 120 received second-line setting. According to the cohorts, the median PFS was 5.7 months (95%CI, 5.4-7.4) and 5.5 months (95% CI, 3.7-5.6), respectively. Although the median OS was considered immature, after estimating the survival curve by kaplan-meier analysis, the 12-month survival rates were 74.7% (95% CI, 62.5-83.5) and 68.2 % (95% CI, 59.0-75.7), for the first-line and second-line cohorts. ORR was 34.3% (24/70; 95% CI, 23.3-46.6) in the first-line and 22.5% (27/120; 95% CI, 15.4-31.0) in the second-line cohort. The trial showed that combination of camrelizumab and apatinib achieved potent efficacy in aHCC both in the first-line and second-line settings.

## Localized treatments in HCC

### Neoadjuvant and adjuvant therapy

The purpose of adjuvant therapy is to help reduce the postoperative recurrence rate of HCC patients. Neoadjuvant therapy can significantly reduce the tumor and inhibit the proliferation and metastasis of tumor cells, so as to improve the clinical symptoms of patients and better accept surgical treatment 172, 173. However, the role of neoadjuvant therapy in the treatment of HCC is still unclear. In fact, there are relatively few studies on the concept of neoadjuvant therapy for HCC 172. In the data we found, neoadjuvant therapy and adjuvant therapy do not appear to be required by the guidelines for the treatment of HCC and there are no FDA approved treatment options for adjuvant or neoadjuvant HCC therapy after resection/ablation with curative intent. The United States Cancer Board defines neoadjuvant therapy as radiotherapy (RT) and systemic therapy 174. Both methods include TACE, radiotherapy and ICIs. In addition, adjuvant therapy included IFN-α, molecular targeted drugs, traditional Chinese medicine, and new adjuvant therapy included systemic therapy, anti-viral therapy, HAIC, transarterial radioembolization (TARE). Although TACE is primarily applied to neoadjuvant therapy, it is also assessed as an adjuvant therapy after resection. The study found that preoperative RT can be used as a bridging therapy in patients with advanced HCC, and adjuvant RT may be superior to TACE in terms of recurrence-free survival (RFS) and OS. ICIs have been approved for adjuvant therapy for other solid tumors, and ICIs may also play a role as a new adjuvant therapy during perioperative period of HCC. In preclinical studies, IFN-α was discovered to reduce the occurrence of tumor recurrence after orthotopic liver transplantation. TKIs have achieved great success in many kinds of solid tumors including HCC. Sorafenib is the first approved TKI for advanced HCC. In a study of sorafenib as adjuvant therapy for HCC, the recurrence rate of patients after hepatectomy treated with sorafenib was significantly lower than that of the control group 175. Traditional Chinese medicine has a long history in China. Huaier granules are widely used in the treatment of various cancers, and have been proved to be able to prolong RFS of HCC patients as adjuvant therapy 176. Systemic therapy, including systemic chemotherapy, drug therapy and immunotherapy, can be used as a new adjuvant therapy to reduce HCC staging 172, 173, 177. In HCC, hepatic artery is the main blood source conducive to tumor growth, so HAIC selectively delivers chemotherapy agents in the liver parenchyma through catheters or pumps to directly attack local diseases 178. TARE is the use of radioactive elements produced by the way of intra-arterial injection of liver cancer radiotherapy, more suitable for HCC patients with portal vein tumor thrombosis.

After considering the established role of PD-1 and CTLA-4 blockers in adjuvant therapy for melanoma patients, especially ipilimumab and pembrolizumab have been approved for melanoma, researchers wondered whether ICI could play a role in neoadjuvant therapy or adjuvant therapy for HCC patients 179-181. Nivolumab and pembrolizumab have been studied as adjuvant therapy for HCC (NCT03383458 and NCT03867084). Currently, many ongoing phase 3 clinical trials for adjuvant therapies evaluate an anti-PD-1 antibody with or without an anti-angiogenic agent, such as adjuvant pembrolizumab (KEYNOTE-937, NCT03867084), adjuvant nivolumab (CheckMate 9DX, NCT03383458), adjuvant durvalumab (anti-PD-L1 antibody) with or without bevacizumab (angiogenesis inhibitor) (EMERALD-2, NCT03847428), adjuvant toripalimab (JS001) (JUPITER 04, NCT03859128) and adjuvant atezolizumab plus bevacizumab (IMbrave050, NCT04102098).

#### Locoregional therapies

HCC usually occurs in chronic inflammatory environments, especially inflammation caused by viral hepatitis. Liver transplantation and surgical resection are the main treatments for patients with early HCC 182. However, when a large number of patients are diagnosed, the disease is already in the middle and late stages or serious complications, which undoubtedly increases the risk of surgery, so it is not suitable for surgical treatment 182. Local treatment has become a new choice for these patients, mainly including transarterial embolization (TAE), TACE, TARE, and ablative therapies 183, 184. Studies have verified that direct killing of tumors can lead to activation of the immune system. However, in recent years, TAE and TARE are rarely used in combination with ICI in HCC-related research. Leuchte K et al. tested the effect of microwave ablation on tumor specific immune response in patients with HCC 185. The researchers analyzed specific phenotypes of T and B cell subsets in 23 patients treated with MWA alone. The results demonstrate that after MWA treatment, most T and B cell subsets did not change significantly, and the frequency of effector memory T cells decreased (23.7% ± 1.6, day 0 28.5% ± 2.3; p < 0.01), while plasma mother cells increased on the 7th day after treatment (8.3% ± 1.7, day 0 4.0% ± 1.2; p = 0.02). They also analyzed whether the specific changes of circulating immune cell subsets in patients with early recurrence and long-term remission were related to MWA. The data revealed that compared with patients with long-term remission, CD69^+^ T cells were enriched in patients with recurrence. T cell depletion marker PD-1 and Tim-3 mediated NK cell tolerance were also upregulated in patients with early recurrence. These results suggested that 30% of patients had increased specific immune response, that is, these patients showed increased TAA-specific IFN-γ or IL-5 secretion after stimulation with HCC-related antigens after MWA. This experiment highlighted the immunogenicity of MWA and was important for detecting immune response and inhibiting anti-tumor immunity in ongoing and future clinical trials of MWA combined with ICIs.

TACE is not a radical treatment, which may lead to poor results such as deterioration of liver function and extrahepatic metastasis 186, 187. Koroki K et al. studied and evaluated the therapeutic effect of TCAE in patients with medium-term HCC 186. Studies had shown that the results of TACE in patients with medium-term HCC were closely related to the number and size of tumors. TACE-induced hypoxia promotes the release of proangiogenic cytokines and the death of immunogenic cells, and promotes tumor angiogenesis and regulates the function of immune cells in tumor microenvironment 188. Therefore, the combination therapy of TACE and sorafenib plus ICIs may have promising therapeutic effect and safety in advanced TACE refractory HCC.

A recent review of the efficacy and safety of TACE combined with sorafenib plus immune checkpoint inhibitors in the treatment of advanced TACE refractory HCC 189. Compared with the TACE + Sor group, the OS and median PFS TACE + Sor + ICIs group were significantly prolonged, and the survival rate of patients was significantly improved. Furthermore, the median PFS of patients receiving nivolumab was similar to that of patients receiving pembrolizumab [13.6 months (95% CI, 12.02-15.18) vs. 13.2 months (95% CI, 6.85-19.55, z = 0.32, P = 0.859)], and as well as the median OS [20.0 months (95% CI, 12.92-27.08) vs. 25.6 months (95% CI, 13.53-37.67, z = 0.05, P = 0.820]. The study demonstrated that TACE combined with sorafenib plus ICIs was effective in patients with advanced refractory HCC.

## Future and Conclusions

ICI has made great progress in the treatment of solid tumors in the past decades, bringing hope for cure of patients. Although the response rate of ICI in HCC patients is not high at present, with the improvement of our understanding of the interaction between ICI and adaptability and innate immune response, more new treatment modes will emerge. Most of the current immunotherapy strategies are to combine ICI with other immune-targeted strategies. And the combination of anti-PD-1+anti-CTLA-4 (nivolumab+ipilimumab) has received FDA's accelerated approval 190. Other immune checkpoints, such as TNFRSF, LAG-3, TIM-3, TIGIT, can regulate T cell function and play an important role in tumor immune escape 91. The anti-tumor effect of ICIs targeting LAG-3, TIM-3 or TIGIT in humans is currently under study. Early trials of combination therapy of anti-TIM-3 and anti-TIGIT antibodies with PD-1 or CTLA-4 in a variety of tumor types are also ongoing and need further exploration 113.

In recent years, platinum-based chemotherapy combined with PD-1/PD-L1 inhibitors has shown efficacy in a variety of cancers, which has gradually become the focus of attention. Studies have found that there are two main therapeutic mechanisms for platinum-based chemotherapy combined with PD-1/PD-L1 inhibitors. Firstly, platinum chemotherapy has a positive effect on immune regulation, which can increase the sensitivity of tumor cells to PD-1/PD-L1 inhibitors 191. On the other hand, higher doses of platinum may increase the expression of PD-L1 in tumor cells but decrease the activity of T cells 191, 192. Strategically combining ICIs with other treatment methods and utilizing their potential synergistic can maximize the clinical activity of combined therapy and establish a lasting immune response, which is crucial to the development of new therapies for cancer.

With the progress of ICI, we are entering a new era of HCC immunotherapy. Immunocytes in HCC tumor immune microenvironment provide new targets and signaling pathways for immunotherapy. These new therapies alleviate the condition of HCC patients and prolong their survival time. Although ICIs still have many disadvantages, such as low response rate, more side effects and complications, many treatments combining ICIs with other therapies (including VEGF or VEGFR inhibition, neoadjuvant and adjuvant therapy, locoregional therapies) have been developed in recent years. Therefore, improving understanding of cancer immunology and how to lead to immune tolerance in HCC is critical to translating findings into clinical outcomes, avoiding unnecessary medications and guiding rational treatment.

## Figures and Tables

**Table 1:** The clinical trials on immune checkpoints and other therapeutic targets for HCC in recent years

ClinicalTrials.govIdentifier	Trial name	Agent	Target	Endpoints	Phase	Actual Enrollment	Recruitment Status
NCT03695250		BMS-986205 vs Nivolumab	IDO1, PD-1	Safety/Tolerability/ORR	1/2	8	Active, not recruiting
NCT01658878	CheckMate040	Nivolumab plus Cabozantinib +/- Ipilimumab	PD-1, CTLA-4, TKI	Safety/Tolerability/ORR	1/2	659	Active, not recruiting
NCT03006926		Pembrolizumab plus Lenvatinib	PD-1, TKI	Safety	1	104	Active, not recruiting
NCT03222076		Nivolumab +/- Ipilimumab	PD-1, CTLA-4	Safety/Tolerability	2	30	Active, not recruiting
NCT03434379	IMbrave150	Atezolizumab plus Bevacizumab vs Sorafenib	PD-L1, VEGFR, TKI	PFS/OS	3	558	Active, not recruiting
NCT03383458	CheckMate 9DX	Nivolumab	PD-1	RFS	3	545	Active, not recruiting
NCT03867084	KEYNOTE-937	Pembrolizumab	PD-1	RFS/OS	3	950	Recruiting
NCT03847428	EMERALD-2	Durvalumab +/- Bevacizumab	PD-L1, VEGFR	RFS	3	888	Recruiting
NCT03859128	JUPITER 04	Toripalimab	PD-1	RFS	2/3	402	Active, not recruiting
NCT04102098	IMbrave050	Atezolizumab plus Bevacizumab	PD-L1, VEGFR	RFS	3	668	Active, not recruiting

**Table 2:** Comparison between rechallenge and nonrechallenge cases after an irAE with at least 1 immune checkpoint inhibitor (n = 24 079)

Initial irAE	No. (%), Rechallenge after irAE (n = 6123)	No rechallenge after irAE (n = 17 956)
**ICI**		
Anti-PD-1 or anti-PD-L1 alone	4360 (71.2)	12 321 (68.6)
Anti-CTLA-4 alone	791 (12.9)	3290 (18.3)
Combination therapy	972 (15.9)	2345 (13.1)
**Type of initial irAE (only five diseases with the highest rechallenge rate were selected)**		
Colitis	1745 (28.5)	5353 (29.8)
Pneumonitis	1288 (21.0)	4001 (22.3)
Thyroiditis	779 (12.7)	1977 (11.0)
Arthritis	491 (8.0)	1372 (7.6)
Hepatitis	473 (7.7)	1444 (8.0)

Abbreviations: CTLA-4, cytotoxic T-lymphocyte antigen-4; ICI, immune checkpoint inhibitor; irAE, immune-related adverse event; PD-1, programmed cell death 1; PD-L1, programmed cell death 1 ligand 1.

## Abbreviations

APC: antigen presenting cells
AFP: alpha fetoprotein
aHCC: advanced hepatocellular carcinoma
BCLC: Barcelona Clinic HCC
BsAb: bispecific antibody
CEACAM-1: carcinoembryonic antigen-related cell adhesion molecule 1
C/EBPβ: CCAAT-Enhancer-Binding Protein Beta
Clec4g: C-type lectin-like domain containing-4g
c-SMAC: central supramolecular cluster
CTLA-4: cytotoxic T lymphocyte 4
CTLs: cytotoxic T lymphocytes
DC: dendritic cells
DCR: disease control rate
DOR: duration of response
DPT: double‐positive T
DRR: durable response rate
EMT: epithelial-mesenchymal transition
ERAD: endoplasmic reticulum-associated degradation
FGL1: fibrinogen-like protein 1
Gal-9: galactose lectin 9
GPC3: Glypican-3
HAIC: hepatic arterial infusion chemotherapy
HAT: hepatic artery infusion pump
HCC: Hepatocellular carcinoma
HMGB1: high mobility group box-1 protein
HR: hepatic resection
HSC: hepatic stellate cells
ICIs: immune checkpoint inhibitors
IDO: 2,3-dioxygenase
Ig: immunoglobulin
IIR: independent imaging review
iNOS: inducible nitric oxide synthase
irAEs: immune-related adverse events
IrAEs-3/4: grade 3 or 4 immune-related adverse events
iTregs: induced-to-adjust T cells
KCs: Kupffer cells
LAG-3: Lymphocyte activating gene 3
MDSCs: myeloid-derived suppressor cells
M-MDSC: mononuclear MDSC
MPS: mononuclear phagocyte system
MHC-II: major histocompatibility complex II
MTM-HCC: macrotrabecular-massive HCC
NIVO: nivolumab
NK cells: natural killer cells
NMA: network meta-analysis
nTregs: natural regulatory T cells
OTUB1: OTU domain-containing ubiquitin aldehyde-binding protein 1
ORR: Objective Response Rate
PD-1: programmed cell death protein 1
PD-L1: programmed cell death ligand 1
PFS: progression-free survival
PMN-MDSC: polymorphonuclear MDSC
PtdSer: phosphatidylserine
RFS: recurrence-free survival
ROS: reactive oxygen species
RT: radiotherapy
SOR: sorafenib
TAA: tumor associated antigens
TACE: transarterial chemoembolization
TAE: transarterial embolization
TAMs: tumor-associated macrophages
TARE: transarterial radioembolization
TCR: T cell receptor
TDO or TDO2: tryptophan 2,3-dioxygenase
TGF-β: transforming growth factor-β
TE: transendocytosis
Th cell: T helper cell
TIGIT: T cell immunoreceptor with immunoglobulin and ITIM domain
TIL: tumor-infiltrating lymphocytes
TIM-3: T cell immunoglobulin and mucin domain-containing protein 3
TKIs: Tyrosine kinase inhibitors
TME: tumor microenvironment
TNFRSF: tumor necrosis factor receptor superfamily
Tregs: regulatory T cells
uHCC: unresectable hepatocellular carcinoma
VEGF: Vascular endothelial-derived growth factor

## References

[1] Sung H, Ferlay J, Siegel RL, Laversanne M, Soerjomataram I, Jemal A (2021). Global Cancer Statistics 2020: GLOBOCAN Estimates of Incidence and Mortality Worldwide for 36 Cancers in 185 Countries. CA Cancer J Clin.

[2] Arnold M, Abnet CC, Neale RE, Vignat J, Giovannucci EL, McGlynn KA (2020). Global Burden of 5 Major Types of Gastrointestinal Cancer. Gastroenterology.

[3] Xie DY, Ren ZG, Zhou J, Fan J, Gao Q (2020). 2019 Chinese clinical guidelines for the management of hepatocellular carcinoma: updates and insights. Hepatobiliary Surg Nutr.

[4] Llovet JM, Kelley RK, Villanueva A, Singal AG, Pikarsky E, Roayaie S (2021). Hepatocellular carcinoma. Nature reviews Disease primers.

[5] Finn RS, Zhu AX (2021). Evolution of Systemic Therapy for Hepatocellular Carcinoma. Hepatology.

[6] Chen Z, Xie H, Hu M, Huang T, Hu Y, Sang N (2020). Recent progress in treatment of hepatocellular carcinoma. American journal of cancer research.

[7] Raoul JL, Forner A, Bolondi L, Cheung TT, Kloeckner R, de Baere T (2019). Updated use of TACE for hepatocellular carcinoma treatment: How and when to use it based on clinical evidence. Cancer treatment reviews.

[8] Couri T, Pillai A (2019). Goals and targets for personalized therapy for HCC. Hepatol Int.

[9] Fu Y, Liu S, Zeng S, Shen H (2019). From bench to bed: the tumor immune microenvironment and current immunotherapeutic strategies for hepatocellular carcinoma. J Exp Clin Cancer Res.

[10] Johnston MP, Khakoo SI (2019). Immunotherapy for hepatocellular carcinoma: Current and future. World J Gastroenterol.

[11] Gao Q, Qiu SJ, Fan J, Zhou J, Wang XY, Xiao YS (2007). Intratumoral balance of regulatory and cytotoxic T cells is associated with prognosis of hepatocellular carcinoma after resection. J Clin Oncol.

[12] Bian J, Lin J, Long J, Yang X, Yang X, Lu X (2020). T lymphocytes in hepatocellular carcinoma immune microenvironment: insights into human immunology and immunotherapy. American journal of cancer research.

[13] Chen Y, Tian Z (2021). Innate lymphocytes: pathogenesis and therapeutic targets of liver diseases and cancer. Cellular & molecular immunology.

[14] Han JW, Yoon SK (2020). Tissue-Resident Lymphocytes: Implications in Immunotherapy for Hepatocellular Carcinoma. Int J Mol Sci.

[15] Klugewitz K, Adams DH, Emoto M, Eulenburg K, Hamann A (2004). The composition of intrahepatic lymphocytes: shaped by selective recruitment?. Trends Immunol.

[16] Cai L, Zhang Z, Zhou L, Wang H, Fu J, Zhang S (2008). Functional impairment in circulating and intrahepatic NK cells and relative mechanism in hepatocellular carcinoma patients. Clin Immunol.

[17] Giraud J, Chalopin D, Blanc JF, Saleh M (2021). Hepatocellular Carcinoma Immune Landscape and the Potential of Immunotherapies. Frontiers in immunology.

[18] Medina-Echeverz J, Eggert T, Han M, Greten TF (2015). Hepatic myeloid-derived suppressor cells in cancer. Cancer immunology, immunotherapy: CII.

[19] Chew V, Chen J, Lee D, Loh E, Lee J, Lim KH (2012). Chemokine-driven lymphocyte infiltration: an early intratumoural event determining long-term survival in resectable hepatocellular carcinoma. Gut.

[20] Zheng Y, Li Y, Lian J, Yang H, Li F, Zhao S (2019). TNF-alpha-induced Tim-3 expression marks the dysfunction of infiltrating natural killer cells in human esophageal cancer. J Transl Med.

[21] Zhang PF, Gao C, Huang XY, Lu JC, Guo XJ, Shi GM (2020). Cancer cell-derived exosomal circUHRF1 induces natural killer cell exhaustion and may cause resistance to anti-PD1 therapy in hepatocellular carcinoma. Mol Cancer.

[22] Tan S, Xu Y, Wang Z, Wang T, Du X, Song X (2020). Tim-3 Hampers Tumor Surveillance of Liver-Resident and Conventional NK Cells by Disrupting PI3K Signaling. Cancer Res.

[23] Steinman RM, Hawiger D, Nussenzweig MC (2003). Tolerogenic dendritic cells. Annu Rev Immunol.

[24] Lurje I, Hammerich L, Tacke F (2020). Dendritic Cell and T Cell Crosstalk in Liver Fibrogenesis and Hepatocarcinogenesis: Implications for Prevention and Therapy of Liver Cancer. Int J Mol Sci.

[25] Ruf B, Heinrich B, Greten TF (2021). Immunobiology and immunotherapy of HCC: spotlight on innate and innate-like immune cells. Cellular & molecular immunology.

[26] Palmer DH, Midgley RS, Mirza N, Torr EE, Ahmed F, Steele JC (2009). A phase II study of adoptive immunotherapy using dendritic cells pulsed with tumor lysate in patients with hepatocellular carcinoma. Hepatology.

[27] Teng CF, Wang T, Wu TH, Lin JH, Shih FY, Shyu WC (2020). Combination therapy with dendritic cell vaccine and programmed death ligand 1 immune checkpoint inhibitor for hepatocellular carcinoma in an orthotopic mouse model. Ther Adv Med Oncol.

[28] Dong Z, Wei H, Sun R, Tian Z (2007). The roles of innate immune cells in liver injury and regeneration. Cellular & molecular immunology.

[29] Kong L, Zhou Y, Bu H, Lv T, Shi Y, Yang J (2016). Deletion of interleukin-6 in monocytes/macrophages suppresses the initiation of hepatocellular carcinoma in mice. J Exp Clin Cancer Res.

[30] Qian BZ, Pollard JW (2010). Macrophage diversity enhances tumor progression and metastasis. Cell.

[31] Sica A, Invernizzi P, Mantovani A (2014). Macrophage plasticity and polarization in liver homeostasis and pathology. Hepatology.

[32] Yang Y, Ye YC, Chen Y, Zhao JL, Gao CC, Han H (2018). Crosstalk between hepatic tumor cells and macrophages via Wnt/beta-catenin signaling promotes M2-like macrophage polarization and reinforces tumor malignant behaviors. Cell Death Dis.

[33] Tian X, Wu Y, Yang Y, Wang J, Niu M, Gao S (2020). Long noncoding RNA LINC00662 promotes M2 macrophage polarization and hepatocellular carcinoma progression via activating Wnt/beta-catenin signaling. Molecular oncology.

[34] He Y, Xu Y, Yu X, Sun Z, Guo W (2021). The Vital Roles of LINC00662 in Human Cancers. Frontiers in cell and developmental biology.

[35] Zhou D, Luan J, Huang C, Li J (2021). Tumor-Associated Macrophages in Hepatocellular Carcinoma: Friend or Foe?. Gut Liver.

[36] Korbecki J, Kojder K, Siminska D, Bohatyrewicz R, Gutowska I, Chlubek D (2020). CC Chemokines in a Tumor: A Review of Pro-Cancer and Anti-Cancer Properties of the Ligands of Receptors CCR1, CCR2, CCR3, and CCR4. Int J Mol Sci.

[37] Liu C, Chikina M, Deshpande R, Menk AV, Wang T, Tabib T (2019). Treg Cells Promote the SREBP1-Dependent Metabolic Fitness of Tumor-Promoting Macrophages via Repression of CD8(+) T Cell-Derived Interferon-gamma. Immunity.

[38] Ormandy LA, Hillemann T, Wedemeyer H, Manns MP, Greten TF, Korangy F (2005). Increased populations of regulatory T cells in peripheral blood of patients with hepatocellular carcinoma. Cancer Res.

[39] Yang Y, Liu F, Liu W, Ma M, Gao J, Lu Y (2020). Analysis of single-cell RNAseq identifies transitional states of T cells associated with hepatocellular carcinoma. Clin Transl Med.

[40] Wing JB, Tanaka A, Sakaguchi S (2019). Human FOXP3(+) Regulatory T Cell Heterogeneity and Function in Autoimmunity and Cancer. Immunity.

[41] Li C, Jiang P, Wei S, Xu X, Wang J (2020). Regulatory T cells in tumor microenvironment: new mechanisms, potential therapeutic strategies and future prospects. Mol Cancer.

[42] Paluskievicz CM, Cao X, Abdi R, Zheng P, Liu Y, Bromberg JS (2019). T Regulatory Cells and Priming the Suppressive Tumor Microenvironment. Frontiers in immunology.

[43] Whiteside TL (2012). What are regulatory T cells (Treg) regulating in cancer and why?. Semin Cancer Biol.

[44] Langhans B, Nischalke HD, Kramer B, Dold L, Lutz P, Mohr R (2019). Role of regulatory T cells and checkpoint inhibition in hepatocellular carcinoma. Cancer immunology, immunotherapy: CII.

[45] Shi C, Chen Y, Chen Y, Yang Y, Bing W, Qi J (2019). CD4(+) CD25(+) regulatory T cells promote hepatocellular carcinoma invasion via TGF-beta1-induced epithelial-mesenchymal transition. Onco Targets Ther.

[46] Sawant DV, Yano H, Chikina M, Zhang Q, Liao M, Liu C (2019). Adaptive plasticity of IL-10(+) and IL-35(+) Treg cells cooperatively promotes tumor T cell exhaustion. Nature immunology.

[47] Gabrilovich DI, Ostrand-Rosenberg S, Bronte V (2012). Coordinated regulation of myeloid cells by tumours. Nat Rev Immunol.

[48] Kalathil SG, Thanavala Y (2021). Importance of myeloid derived suppressor cells in cancer from a biomarker perspective. Cell Immunol.

[49] Liu M, Zhou J, Liu X, Feng Y, Yang W, Wu F (2020). Targeting monocyte-intrinsic enhancer reprogramming improves immunotherapy efficacy in hepatocellular carcinoma. Gut.

[50] Veglia F, Perego M, Gabrilovich D (2018). Myeloid-derived suppressor cells coming of age. Nature immunology.

[51] Kumar V, Patel S, Tcyganov E, Gabrilovich DI (2016). The Nature of Myeloid-Derived Suppressor Cells in the Tumor Microenvironment. Trends Immunol.

[52] Hsieh CC, Hung CH, Chiang M, Tsai YC, He JT (2019). Hepatic Stellate Cells Enhance Liver Cancer Progression by Inducing Myeloid-Derived Suppressor Cells through Interleukin-6 Signaling. Int J Mol Sci.

[53] Tian X, Shen H, Li Z, Wang T, Wang S (2019). Tumor-derived exosomes, myeloid-derived suppressor cells, and tumor microenvironment. Journal of hematology & oncology.

[54] Wang Y, Zhang T, Sun M, Ji X, Xie M, Huang W (2021). Therapeutic Values of Myeloid-Derived Suppressor Cells in Hepatocellular Carcinoma: Facts and Hopes. Cancers (Basel).

[55] Law AMK, Valdes-Mora F, Gallego-Ortega D (2020). Myeloid-Derived Suppressor Cells as a Therapeutic Target for Cancer. Cells.

[56] Dong Y, Liu TH, Yau T, Hsu C (2020). Novel systemic therapy for hepatocellular carcinoma. Hepatol Int.

[57] Dermani FK, Samadi P, Rahmani G, Kohlan AK, Najafi R (2019). PD-1/PD-L1 immune checkpoint: Potential target for cancer therapy. Journal of cellular physiology.

[58] Qin S, Xu L, Yi M, Yu S, Wu K, Luo S (2019). Novel immune checkpoint targets: moving beyond PD-1 and CTLA-4. Mol Cancer.

[59] Kalbasi A, Ribas A (2020). Tumour-intrinsic resistance to immune checkpoint blockade. Nat Rev Immunol.

[60] Giannone G, Ghisoni E, Genta S, Scotto G, Tuninetti V, Turinetto M (2020). Immuno-Metabolism and Microenvironment in Cancer: Key Players for Immunotherapy. Int J Mol Sci.

[61] Akinleye A, Rasool Z (2019). Immune checkpoint inhibitors of PD-L1 as cancer therapeutics. Journal of hematology & oncology.

[62] Li B, Chan HL, Chen P (2019). Immune Checkpoint Inhibitors: Basics and Challenges. Curr Med Chem.

[63] Salmaninejad A, Valilou SF, Shabgah AG, Aslani S, Alimardani M, Pasdar A (2019). PD-1/PD-L1 pathway: Basic biology and role in cancer immunotherapy. Journal of cellular physiology.

[64] Zhang X, Schwartz JC, Guo X, Bhatia S, Cao E, Lorenz M (2004). Structural and functional analysis of the costimulatory receptor programmed death-1. Immunity.

[65] Constantinidou A, Alifieris C, Trafalis DT (2019). Targeting Programmed Cell Death -1 (PD-1) and Ligand (PD-L1): A new era in cancer active immunotherapy. Pharmacol Ther.

[66] Patsoukis N, Wang Q, Strauss L, Boussiotis VA (2020). Revisiting the PD-1 pathway. Sci Adv.

[67] Kotanides H, Li Y, Malabunga M, Carpenito C, Eastman SW, Shen Y (2020). Bispecific Targeting of PD-1 and PD-L1 Enhances T-cell Activation and Antitumor Immunity. Cancer Immunol Res.

[68] Wu X, Gu Z, Chen Y, Chen B, Chen W, Weng L (2019). Application of PD-1 Blockade in Cancer Immunotherapy. Computational and structural biotechnology journal.

[69] Gou Q, Dong C, Xu H, Khan B, Jin J, Liu Q (2020). PD-L1 degradation pathway and immunotherapy for cancer. Cell Death Dis.

[70] Tanegashima T, Togashi Y, Azuma K, Kawahara A, Ideguchi K, Sugiyama D (2019). Immune Suppression by PD-L2 against Spontaneous and Treatment-Related Antitumor Immunity. Clin Cancer Res.

[71] Latchman Y, Wood CR, Chernova T, Chaudhary D, Borde M, Chernova I (2001). PD-L2 is a second ligand for PD-1 and inhibits T cell activation. Nature immunology.

[72] Wherry EJ (2011). T cell exhaustion. Nature immunology.

[73] Bonorino C, Mognol G (2020). Editorial: T Cell Exhaustion. Frontiers in immunology.

[74] Blank CU, Haining WN, Held W, Hogan PG, Kallies A, Lugli E (2019). Defining 'T cell exhaustion'. Nat Rev Immunol.

[75] Wolf Y, Anderson AC, Kuchroo VK (2020). TIM3 comes of age as an inhibitory receptor. Nat Rev Immunol.

[76] Ma J, Zheng B, Goswami S, Meng L, Zhang D, Cao C (2019). PD1(Hi) CD8(+) T cells correlate with exhausted signature and poor clinical outcome in hepatocellular carcinoma. Journal for immunotherapy of cancer.

[77] Dammeijer F, van Gulijk M, Mulder EE, Lukkes M, Klaase L, van den Bosch T (2020). The PD-1/PD-L1-Checkpoint Restrains T cell Immunity in Tumor-Draining Lymph Nodes. Cancer Cell.

[78] Zhou X, Yu J, Cheng X, Zhao B, Manyam GC, Zhang L (2019). The deubiquitinase Otub1 controls the activation of CD8(+) T cells and NK cells by regulating IL-15-mediated priming. Nature immunology.

[79] Zhu D, Xu R, Huang X, Tang Z, Tian Y, Zhang J (2021). Deubiquitinating enzyme OTUB1 promotes cancer cell immunosuppression via preventing ER-associated degradation of immune checkpoint protein PD-L1. Cell Death Differ.

[80] Huggins MA, Hamilton SE (2019). Deubiquitinase Otub1 negatively regulates IL-15 signaling in CD8 T cells and NK cells. Cellular & molecular immunology.

[81] Lei Q, Wang D, Sun K, Wang L, Zhang Y (2020). Resistance Mechanisms of Anti-PD1/PDL1 Therapy in Solid Tumors. Frontiers in cell and developmental biology.

[82] Nakayama Y, Mimura K, Tamaki T, Shiraishi K, Kua LF, Koh V (2019). PhosphoSTAT1 expression as a potential biomarker for antiPD1/antiPDL1 immunotherapy for breast cancer. Int J Oncol.

[83] Alspach E, Lussier DM, Schreiber RD (2019). Interferon gamma and Its Important Roles in Promoting and Inhibiting Spontaneous and Therapeutic Cancer Immunity. Cold Spring Harb Perspect Biol.

[84] Su D, Tsai HI, Xu Z, Yan F, Wu Y, Xiao Y (2019). Exosomal PD-L1 functions as an immunosuppressant to promote wound healing. J Extracell Vesicles.

[85] Poggio M, Hu T, Pai CC, Chu B, Belair CD, Chang A (2019). Suppression of Exosomal PD-L1 Induces Systemic Anti-tumor Immunity and Memory. Cell.

[86] Zheng B, Wang D, Qiu X, Luo G, Wu T, Yang S (2020). Trajectory and Functional Analysis of PD-1(high) CD4(+)CD8(+) T Cells in Hepatocellular Carcinoma by Single-Cell Cytometry and Transcriptome Sequencing. Adv Sci (Weinh).

[87] Tobin JWD, Keane C, Gunawardana J, Mollee P, Birch S, Hoang T (2019). Progression of Disease Within 24 Months in Follicular Lymphoma Is Associated With Reduced Intratumoral Immune Infiltration. J Clin Oncol.

[88] Solinas C, Aiello M, Rozali E, Lambertini M, Willard-Gallo K, Migliori E (2020). Programmed cell death-ligand 2: A neglected but important target in the immune response to cancer?. Transl Oncol.

[89] Zhang Y, Xu J, Hua J, Liu J, Liang C, Meng Q (2019). A PD-L2-based immune marker signature helps to predict survival in resected pancreatic ductal adenocarcinoma. Journal for immunotherapy of cancer.

[90] Yasuoka H, Asai A, Ohama H, Tsuchimoto Y, Fukunishi S, Higuchi K (2020). Increased both PD-L1 and PD-L2 expressions on monocytes of patients with hepatocellular carcinoma was associated with a poor prognosis. Sci Rep.

[91] Xu W, Atkins MB, McDermott DF (2020). Checkpoint inhibitor immunotherapy in kidney cancer. Nat Rev Urol.

[92] Mitsuiki N, Schwab C, Grimbacher B (2019). What did we learn from CTLA-4 insufficiency on the human immune system?. Immunol Rev.

[93] Bao S, Jiang X, Jin S, Tu P, Lu J (2021). TGF-beta1 Induces Immune Escape by Enhancing PD-1 and CTLA-4 Expression on T Lymphocytes in Hepatocellular Carcinoma. Front Oncol.

[94] Linsley PS, Brady W, Grosmaire L, Aruffo A, Damle NK, Ledbetter JA (1991). Binding of the B cell activation antigen B7 to CD28 costimulates T cell proliferation and interleukin 2 mRNA accumulation. J Exp Med.

[95] Liu Y, Zheng P (2020). Preserving the CTLA-4 Checkpoint for Safer and More Effective Cancer Immunotherapy. Trends Pharmacol Sci.

[96] Chen R, Ganesan A, Okoye I, Arutyunova E, Elahi S, Lemieux MJ (2020). Targeting B7-1 in immunotherapy. Med Res Rev.

[97] Oyewole-Said D, Konduri V, Vazquez-Perez J, Weldon SA, Levitt JM, Decker WK (2020). Beyond T-Cells: Functional Characterization of CTLA-4 Expression in Immune and Non-Immune Cell Types. Frontiers in immunology.

[98] Hosseini A, Gharibi T, Marofi F, Babaloo Z, Baradaran B (2020). CTLA-4: From mechanism to autoimmune therapy. Int Immunopharmacol.

[99] Ovcinnikovs V, Ross EM, Petersone L, Edner NM, Heuts F, Ntavli E (2019). CTLA-4-mediated transendocytosis of costimulatory molecules primarily targets migratory dendritic cells. Sci Immunol.

[100] Yang Y, Li X, Ma Z, Wang C, Yang Q, Byrne-Steele M (2021). CTLA-4 expression by B-1a B cells is essential for immune tolerance. Nat Commun.

[101] Kvarnhammar AM, Veitonmaki N, Hagerbrand K, Dahlman A, Smith KE, Fritzell S (2019). The CTLA-4 x OX40 bispecific antibody ATOR-1015 induces anti-tumor effects through tumor-directed immune activation. Journal for immunotherapy of cancer.

[102] Dostert C, Grusdat M, Letellier E, Brenner D (2019). The TNF Family of Ligands and Receptors: Communication Modules in the Immune System and Beyond. Physiol Rev.

[103] Buchan SL, Dou L, Remer M, Booth SG, Dunn SN, Lai C (2018). Antibodies to Costimulatory Receptor 4-1BB Enhance Anti-tumor Immunity via T Regulatory Cell Depletion and Promotion of CD8 T Cell Effector Function. Immunity.

[104] Sun Y, Lin X, Chen HM, Wu Q, Subudhi SK, Chen L (2002). Administration of agonistic anti-4-1BB monoclonal antibody leads to the amelioration of experimental autoimmune encephalomyelitis. J Immunol.

[105] Watts TH (2005). TNF/TNFR family members in costimulation of T cell responses. Annu Rev Immunol.

[106] Remedios KA, Zirak B, Sandoval PM, Lowe MM, Boda D, Henley E (2018). The TNFRSF members CD27 and OX40 coordinately limit TH17 differentiation in regulatory T cells. Sci Immunol.

[107] Lubrano di Ricco M, Ronin E, Collares D, Divoux J, Gregoire S, Wajant H (2020). Tumor necrosis factor receptor family costimulation increases regulatory T-cell activation and function via NF-kappaB. Eur J Immunol.

[108] Starzer AM, Berghoff AS (2020). New emerging targets in cancer immunotherapy: CD27 (TNFRSF7). ESMO Open.

[109] Gaspar M, Pravin J, Rodrigues L, Uhlenbroich S, Everett KL, Wollerton F (2020). CD137/OX40 Bispecific Antibody Induces Potent Antitumor Activity that Is Dependent on Target Coengagement. Cancer Immunol Res.

[110] Chauvin JM, Zarour HM (2020). TIGIT in cancer immunotherapy. Journal for immunotherapy of cancer.

[111] Khan M, Arooj S, Wang H (2020). NK Cell-Based Immune Checkpoint Inhibition. Frontiers in immunology.

[112] Ge Z, Zhou G, Campos Carrascosa L, Gausvik E, Boor PPC, Noordam L (2021). TIGIT and PD1 Co-blockade Restores *ex vivo* Functions of Human Tumor-Infiltrating CD8(+) T Cells in Hepatocellular Carcinoma. Cell Mol Gastroenterol Hepatol.

[113] Zheng Q, Xu J, Gu X, Wu F, Deng J, Cai X (2020). Immune checkpoint targeting TIGIT in hepatocellular carcinoma. Am J Transl Res.

[114] Kucan Brlic P, Lenac Rovis T, Cinamon G, Tsukerman P, Mandelboim O, Jonjic S (2019). Targeting PVR (CD155) and its receptors in anti-tumor therapy. Cellular & molecular immunology.

[115] Zhang C, Wang Y, Xun X, Wang S, Xiang X, Hu S (2020). TIGIT Can Exert Immunosuppressive Effects on CD8+ T Cells by the CD155/TIGIT Signaling Pathway for Hepatocellular Carcinoma *In vitro*. J Immunother.

[116] O'Donnell JS, Madore J, Li XY, Smyth MJ (2020). Tumor intrinsic and extrinsic immune functions of CD155. Semin Cancer Biol.

[117] Duan X, Liu J, Cui J, Ma B, Zhou Q, Yang X (2019). Expression of TIGIT/CD155 and correlations with clinical pathological features in human hepatocellular carcinoma. Mol Med Rep.

[118] Acharya N, Sabatos-Peyton C, Anderson AC (2020). Tim-3 finds its place in the cancer immunotherapy landscape. Journal for immunotherapy of cancer.

[119] Blackburn SD, Shin H, Freeman GJ, Wherry EJ (2008). Selective expansion of a subset of exhausted CD8 T cells by alphaPD-L1 blockade. Proc Natl Acad Sci U S A.

[120] Kurachi M (2019). CD8(+) T cell exhaustion. Semin Immunopathol.

[121] Kashio Y, Nakamura K, Abedin MJ, Seki M, Nishi N, Yoshida N (2003). Galectin-9 induces apoptosis through the calcium-calpain-caspase-1 pathway. J Immunol.

[122] Zhu C, Anderson AC, Schubart A, Xiong H, Imitola J, Khoury SJ (2005). The Tim-3 ligand galectin-9 negatively regulates T helper type 1 immunity. Nature immunology.

[123] Gleason MK, Lenvik TR, McCullar V, Felices M, O'Brien MS, Cooley SA (2012). Tim-3 is an inducible human natural killer cell receptor that enhances interferon gamma production in response to galectin-9. Blood.

[124] Nakayama M, Akiba H, Takeda K, Kojima Y, Hashiguchi M, Azuma M (2009). Tim-3 mediates phagocytosis of apoptotic cells and cross-presentation. Blood.

[125] Chiba S, Baghdadi M, Akiba H, Yoshiyama H, Kinoshita I, Dosaka-Akita H (2012). Tumor-infiltrating DCs suppress nucleic acid-mediated innate immune responses through interactions between the receptor TIM-3 and the alarmin HMGB1. Nature immunology.

[126] De Sousa Linhares A, Kellner F, Jutz S, Zlabinger GJ, Gabius HJ, Huppa JB (2020). TIM-3 and CEACAM1 do not interact in cis and in trans. Eur J Immunol.

[127] Zhang H, Song Y, Yang H, Liu Z, Gao L, Liang X (2018). Tumor cell-intrinsic Tim-3 promotes liver cancer via NF-kappaB/IL-6/STAT3 axis. Oncogene.

[128] Ganjalikhani Hakemi M, Jafarinia M, Azizi M, Rezaeepoor M, Isayev O, Bazhin AV (2020). The Role of TIM-3 in Hepatocellular Carcinoma: A Promising Target for Immunotherapy?. Front Oncol.

[129] Yang H, Shao R, Huang H, Wang X, Rong Z, Lin Y (2019). Engineering macrophages to phagocytose cancer cells by blocking the CD47/SIRPa axis. Cancer Med.

[130] Chen J, Zheng DX, Yu XJ, Sun HW, Xu YT, Zhang YJ (2019). Macrophages induce CD47 upregulation via IL-6 and correlate with poor survival in hepatocellular carcinoma patients. Oncoimmunology.

[131] Du K, Li Y, Liu J, Chen W, Wei Z, Luo Y (2021). A bispecific antibody targeting GPC3 and CD47 induced enhanced antitumor efficacy against dual antigen-expressing HCC. Mol Ther.

[132] Du J, Wan Z, Wang C, Lu F, Wei M, Wang D (2021). Designer exosomes for targeted and efficient ferroptosis induction in cancer via chemo-photodynamic therapy. Theranostics.

[133] Lemos H, Huang L, Prendergast GC, Mellor AL (2019). Immune control by amino acid catabolism during tumorigenesis and therapy. Nature reviews Cancer.

[134] Li L, Wang T, Li S, Chen Z, Wu J, Cao W (2020). TDO2 Promotes the EMT of Hepatocellular Carcinoma Through Kyn-AhR Pathway. Front Oncol.

[135] Chinnadurai R, Scandolara R, Alese OB, Arafat D, Ravindranathan D, Farris AB (2020). Correlation Patterns Among B7 Family Ligands and Tryptophan Degrading Enzymes in Hepatocellular Carcinoma. Front Oncol.

[136] Prendergast GC, Malachowski WJ, Mondal A, Scherle P, Muller AJ (2018). Indoleamine 2,3-Dioxygenase and Its Therapeutic Inhibition in Cancer. Int Rev Cell Mol Biol.

[137] Andrews LP, Marciscano AE, Drake CG, Vignali DA (2017). LAG3 (CD223) as a cancer immunotherapy target. Immunol Rev.

[138] Wang J, Sanmamed MF, Datar I, Su TT, Ji L, Sun J (2019). Fibrinogen-like Protein 1 Is a Major Immune Inhibitory Ligand of LAG-3. Cell.

[139] Kizuka Y, Kitazume S, Sato K, Taniguchi N (2015). Clec4g (LSECtin) interacts with BACE1 and suppresses Abeta generation. FEBS Lett.

[140] Stillman BN, Hsu DK, Pang M, Brewer CF, Johnson P, Liu FT (2006). Galectin-3 and galectin-1 bind distinct cell surface glycoprotein receptors to induce T cell death. J Immunol.

[141] Guo M, Yuan F, Qi F, Sun J, Rao Q, Zhao Z (2020). Expression and clinical significance of LAG-3, FGL1, PD-L1 and CD8(+)T cells in hepatocellular carcinoma using multiplex quantitative analysis. J Transl Med.

[142] Wang J, Wei W, Tang Q, Lu L, Luo Z, Li W (2020). Oxysophocarpine suppresses hepatocellular carcinoma growth and sensitizes the therapeutic blockade of anti-Lag-3 via reducing FGL1 expression. Cancer Med.

[143] El-Khoueiry AB, Sangro B, Yau T, Crocenzi TS, Kudo M, Hsu C (2017). Nivolumab in patients with advanced hepatocellular carcinoma (CheckMate 040): an open-label, non-comparative, phase 1/2 dose escalation and expansion trial. Lancet.

[144] De Mattia E, Cecchin E, Guardascione M, Foltran L, Di Raimo T, Angelini F (2019). Pharmacogenetics of the systemic treatment in advanced hepatocellular carcinoma. World J Gastroenterol.

[145] Yau T, Park JW, Finn RS, Cheng AL, Mathurin P, Edeline J (2022). Nivolumab versus sorafenib in advanced hepatocellular carcinoma (CheckMate 459): a randomised, multicentre, open-label, phase 3 trial. Lancet Oncol.

[146] Choi WM, Lee D, Shim JH, Kim KM, Lim YS, Lee HC (2020). Effectiveness and Safety of Nivolumab in Child-Pugh B Patients with Hepatocellular Carcinoma: A Real-World Cohort Study. Cancers (Basel).

[147] Kudo M, Matilla A, Santoro A, Melero I, Gracian AC, Acosta-Rivera M (2021). CheckMate 040 Cohort 5: A phase I/II study of nivolumab in patients with advanced hepatocellular carcinoma and Child-Pugh B cirrhosis. Journal of hepatology.

[148] Finn RS, Ryoo BY, Merle P, Kudo M, Bouattour M, Lim HY (2020). Pembrolizumab As Second-Line Therapy in Patients With Advanced Hepatocellular Carcinoma in KEYNOTE-240: A Randomized, Double-Blind, Phase III Trial. J Clin Oncol.

[149] Finn RS, Ikeda M, Zhu AX, Sung MW, Baron AD, Kudo M (2020). Phase Ib Study of Lenvatinib Plus Pembrolizumab in Patients With Unresectable Hepatocellular Carcinoma. J Clin Oncol.

[150] Zhu AX, Finn RS, Edeline J, Cattan S, Ogasawara S, Palmer D (2018). Pembrolizumab in patients with advanced hepatocellular carcinoma previously treated with sorafenib (KEYNOTE-224): a non-randomised, open-label phase 2 trial. Lancet Oncol.

[151] Yau T, Kang YK, Kim TY, El-Khoueiry AB, Santoro A, Sangro B (2020). Efficacy and Safety of Nivolumab Plus Ipilimumab in Patients With Advanced Hepatocellular Carcinoma Previously Treated With Sorafenib: The CheckMate 040 Randomized Clinical Trial. JAMA Oncol.

[152] Kaseb AO, Hasanov E, Cao HST, Xiao L, Vauthey JN, Lee SS (2022). Perioperative nivolumab monotherapy versus nivolumab plus ipilimumab in resectable hepatocellular carcinoma: a randomised, open-label, phase 2 trial. Lancet Gastroenterol Hepatol.

[153] Wong JSL, Kwok GGW, Tang V, Li BCW, Leung R, Chiu J (2021). Ipilimumab and nivolumab/pembrolizumab in advanced hepatocellular carcinoma refractory to prior immune checkpoint inhibitors. Journal for immunotherapy of cancer.

[154] Darnell EP, Mooradian MJ, Baruch EN, Yilmaz M, Reynolds KL (2020). Immune-Related Adverse Events (irAEs): Diagnosis, Management, and Clinical Pearls. Curr Oncol Rep.

[155] Dolladille C, Ederhy S, Sassier M, Cautela J, Thuny F, Cohen AA (2020). Immune Checkpoint Inhibitor Rechallenge After Immune-Related Adverse Events in Patients With Cancer. JAMA Oncol.

[156] Dougan M, Pietropaolo M (2020). Time to dissect the autoimmune etiology of cancer antibody immunotherapy. The Journal of clinical investigation.

[157] Monge C, Xie C, Steinberg SM, Greten TF (2021). Clinical Indicators for Long-Term Survival with Immune Checkpoint Therapy in Advanced Hepatocellular Carcinoma. J Hepatocell Carcinoma.

[158] Hilmi M, Neuzillet C, Calderaro J, Lafdil F, Pawlotsky JM, Rousseau B (2019). Angiogenesis and immune checkpoint inhibitors as therapies for hepatocellular carcinoma: current knowledge and future research directions. Journal for immunotherapy of cancer.

[159] Morse MA, Sun W, Kim R, He AR, Abada PB, Mynderse M (2019). The Role of Angiogenesis in Hepatocellular Carcinoma. Clin Cancer Res.

[160] Zhu XD, Tang ZY, Sun HC (2020). Targeting angiogenesis for liver cancer: Past, present, and future. Genes Dis.

[161] Calderaro J, Ziol M, Paradis V, Zucman-Rossi J (2019). Molecular and histological correlations in liver cancer. Journal of hepatology.

[162] Bourhis M, Palle J, Galy-Fauroux I, Terme M (2021). Direct and Indirect Modulation of T Cells by VEGF-A Counteracted by Anti-Angiogenic Treatment. Frontiers in immunology.

[163] Xia S, Pan Y, Liang Y, Xu J, Cai X (2020). The microenvironmental and metabolic aspects of sorafenib resistance in hepatocellular carcinoma. EBioMedicine.

[164] Cheng AL, Hsu C, Chan SL, Choo SP, Kudo M (2020). Challenges of combination therapy with immune checkpoint inhibitors for hepatocellular carcinoma. Journal of hepatology.

[165] Kudo M (2019). Targeted and immune therapies for hepatocellular carcinoma: Predictions for 2019 and beyond. World J Gastroenterol.

[166] Shigeta K, Datta M, Hato T, Kitahara S, Chen IX, Matsui A (2020). Dual Programmed Death Receptor-1 and Vascular Endothelial Growth Factor Receptor-2 Blockade Promotes Vascular Normalization and Enhances Antitumor Immune Responses in Hepatocellular Carcinoma. Hepatology.

[167] Yi M, Jiao D, Qin S, Chu Q, Wu K, Li A (2019). Synergistic effect of immune checkpoint blockade and anti-angiogenesis in cancer treatment. Mol Cancer.

[168] Torrens L, Montironi C, Puigvehi M, Mesropian A, Leslie J, Haber PK (2021). Immunomodulatory effects of lenvatinib plus anti-PD1 in mice and rationale for patient enrichment in hepatocellular carcinoma. Hepatology.

[169] Sonbol MB, Riaz IB, Naqvi SAA, Almquist DR, Mina S, Almasri J (2020). Systemic Therapy and Sequencing Options in Advanced Hepatocellular Carcinoma: A Systematic Review and Network Meta-analysis. JAMA Oncol.

[170] Finn RS, Qin S, Ikeda M, Galle PR, Ducreux M, Kim TY (2020). Atezolizumab plus Bevacizumab in Unresectable Hepatocellular Carcinoma. N Engl J Med.

[171] Xu J, Shen J, Gu S, Zhang Y, Wu L, Wu J (2021). Camrelizumab in Combination with Apatinib in Patients with Advanced Hepatocellular Carcinoma (RESCUE): A Nonrandomized, Open-label, Phase II Trial. Clin Cancer Res.

[172] Akateh C, Black SM, Conteh L, Miller ED, Noonan A, Elliott E (2019). Neoadjuvant and adjuvant treatment strategies for hepatocellular carcinoma. World J Gastroenterol.

[173] Zhang T, Zhang L, Xu Y, Lu X, Zhao H, Yang H (2020). Neoadjuvant therapy and immunotherapy strategies for hepatocellular carcinoma. American journal of cancer research.

[174] Sahin IH, Khalil L, Millett R, Kaseb A (2021). Neoadjuvant and adjuvant treatment approaches for hepatocellular carcinoma: future outlook. Chin Clin Oncol.

[175] Wang SN, Chuang SC, Lee KT (2014). Efficacy of sorafenib as adjuvant therapy to prevent early recurrence of hepatocellular carcinoma after curative surgery: A pilot study. Hepatol Res.

[176] Chen Q, Shu C, Laurence AD, Chen Y, Peng BG, Zhen ZJ (2018). Effect of Huaier granule on recurrence after curative resection of HCC: a multicentre, randomised clinical trial. Gut.

[177] Zhu XD, Li KS, Sun HC (2020). Adjuvant therapies after curative treatments for hepatocellular carcinoma: Current status and prospects. Genes Dis.

[178] Laface C, Laforgia M, Molinari P, Ugenti I, Gadaleta CD, Porta C (2021). Hepatic Arterial Infusion of Chemotherapy for Advanced Hepatobiliary Cancers: State of the Art. Cancers (Basel).

[179] Ascierto PA, Del Vecchio M, Mandala M, Gogas H, Arance AM, Dalle S (2020). Adjuvant nivolumab versus ipilimumab in resected stage IIIB-C and stage IV melanoma (CheckMate 238): 4-year results from a multicentre, double-blind, randomised, controlled, phase 3 trial. Lancet Oncol.

[180] Tarhini AA, Lee SJ, Hodi FS, Rao UNM, Cohen GI, Hamid O (2020). Phase III Study of Adjuvant Ipilimumab (3 or 10 mg/kg) Versus High-Dose Interferon Alfa-2b for Resected High-Risk Melanoma: North American Intergroup E1609. J Clin Oncol.

[181] Zimmer L, Livingstone E, Hassel JC, Fluck M, Eigentler T, Loquai C (2020). Adjuvant nivolumab plus ipilimumab or nivolumab monotherapy versus placebo in patients with resected stage IV melanoma with no evidence of disease (IMMUNED): a randomised, double-blind, placebo-controlled, phase 2 trial. Lancet.

[182] Villanueva A (2019). Hepatocellular Carcinoma. N Engl J Med.

[183] Inchingolo R, Posa A, Mariappan M, Spiliopoulos S (2019). Locoregional treatments for hepatocellular carcinoma: Current evidence and future directions. World J Gastroenterol.

[184] Makary MS, Khandpur U, Cloyd JM, Mumtaz K, Dowell JD (2020). Locoregional Therapy Approaches for Hepatocellular Carcinoma: Recent Advances and Management Strategies. Cancers (Basel).

[185] Leuchte K, Staib E, Thelen M, Godel P, Lechner A, Zentis P (2021). Microwave ablation enhances tumor-specific immune response in patients with hepatocellular carcinoma. Cancer immunology, immunotherapy: CII.

[186] Koroki K, Ogasawara S, Ooka Y, Kanzaki H, Kanayama K, Maruta S (2020). Analyses of Intermediate-Stage Hepatocellular Carcinoma Patients Receiving Transarterial Chemoembolization prior to Designing Clinical Trials. Liver Cancer.

[187] Chang Y, Jeong SW, Young Jang J, Jae Kim Y (2020). Recent Updates of Transarterial Chemoembolilzation in Hepatocellular Carcinoma. Int J Mol Sci.

[188] El Dika I, Khalil DN, Abou-Alfa GK (2019). Immune checkpoint inhibitors for hepatocellular carcinoma. Cancer.

[189] Zheng L, Fang S, Wu F, Chen W, Chen M, Weng Q (2020). Efficacy and Safety of TACE Combined With Sorafenib Plus Immune Checkpoint Inhibitors for the Treatment of Intermediate and Advanced TACE-Refractory Hepatocellular Carcinoma: A Retrospective Study. Front Mol Biosci.

[190] Wright K (2020). FDA Approves Nivolumab Plus Ipilimumab for the Treatment of Advanced HCC. Oncology (Williston Park).

[191] Xue Y, Gao S, Gou J, Yin T, He H, Wang Y (2021). Platinum-based chemotherapy in combination with PD-1/PD-L1 inhibitors: preclinical and clinical studies and mechanism of action. Expert Opin Drug Deliv.

[192] Park SJ, Ye W, Xiao R, Silvin C, Padget M, Hodge JW (2019). Cisplatin and oxaliplatin induce similar immunogenic changes in preclinical models of head and neck cancer. Oral Oncol.

